# Clinical-grade human skin-derived ABCB5+ mesenchymal stromal cells exert anti-apoptotic and anti-inflammatory effects *in vitro* and modulate mRNA expression in a cisplatin-induced kidney injury murine model

**DOI:** 10.3389/fimmu.2023.1228928

**Published:** 2024-01-11

**Authors:** Erika Rendra, Adriana Torres Crigna, Cristina Daniele, Carsten Sticht, Maike Cueppers, Mark Andreas Kluth, Christoph Ganss, Markus H. Frank, Norbert Gretz, Karen Bieback

**Affiliations:** ^1^Institute of Transfusion Medicine and Immunology, Medical Faculty Mannheim, Heidelberg University, German Red Cross Blood Service Baden-Württemberg - Hessen, Mannheim, Germany; ^2^Medical Faculty Mannheim, Medical Research Centre, Heidelberg University, Mannheim, Germany; ^3^RHEACELL GmbH & Co. KG, Heidelberg, Germany; ^4^Transplant Research Program, Boston Children’s Hospital, Harvard Medical School, Boston, MA, United States; ^5^Harvard Stem Cell Institute, Harvard University, Cambridge, MA, United States; ^6^Harvard Skin Disease Research Center, Department of Dermatology, Brigham and Women’s Hospital, Boston, MA, United States; ^7^School of Medical and Health Sciences, Edith Cowan University, Perth, WA, Australia; ^8^Mannheim Institute for Innate Immunoscience, Medical Faculty Mannheim, Heidelberg University, Mannheim, Germany

**Keywords:** mesenchymal stromal cells, ABCB5, cisplatin, kidney injury, PBMC, macrophage, immune response, mRNA expression profile

## Abstract

Acute kidney injury (AKI) is characterized by a rapid reduction in renal function and glomerular filtration rate (GFR). The broadly used anti-cancer chemotherapeutic agent cisplatin often induces AKI as an adverse drug side effect. Therapies targeted at the reversal of AKI and its potential progression to chronic kidney disease or end-stage renal disease are currently insufficiently effective. Mesenchymal stromal cells (MSCs) possess diverse immunomodulatory properties that confer upon them significant therapeutic potential for the treatment of diverse inflammatory disorders. Human dermal MSCs expressing ATP-Binding Cassette member B5 (ABCB5) have shown therapeutic efficacy in clinical trials in chronic skin wounds or recessive dystrophic epidermolysis bullosa. In preclinical studies, ABCB5+ MSCs have also shown to reverse metabolic reprogramming in polycystic kidney cells, suggesting a capacity for this cell subset to improve also organ function in kidney diseases. Here, we aimed to explore the therapeutic capacity of ABCB5+ MSCs to improve renal function in a preclinical rat model of cisplatin-induced AKI. First, the anti-apoptotic and immunomodulatory capacity was compared against research-grade adipose stromal cells (ASCs). Then, cross-species immunomodulatory capacity was checked, testing first inhibition of mitogen-driven peripheral blood mononuclear cells and then modulation of macrophage function. Finally, therapeutic efficacy was evaluated in a cisplatin AKI model. First, ABCB5+ MSCs suppressed cisplatin-induced apoptosis of human conditionally-immortalized proximal tubular epithelial cells *in vitro*, most likely by reducing oxidative stress. Second, ABCB5+ MSCs inhibited the proliferation of either human or rat peripheral blood mononuclear cells, in the human system via the Indoleamine/kynurenine axis and in the murine context via nitric oxide/nitrite. Third, ABCB5+ MSCs decreased TNF-α secretion after lipopolysaccharide stimulation and modulated phagocytosis and in both human and rat macrophages, involving prostaglandin E2 and TGF-β1, respectively. Fourth, clinical-grade ABCB5+ MSCs grafted intravenously and intraperitoneally to a cisplatin-induced AKI murine model exerted modulatory effects on mRNA expression patterns toward an anti-inflammatory and pro-regenerative state despite an apparent lack of amelioration of renal damage at physiologic, metabolic, and histologic levels. Our results demonstrate anti-inflammatory and pro-regenerative effects of clinical grade ABCB5+ MSCs *in vitro* and *in vivo* and suggest potential therapeutic utility of this cell population for treatment or prevention of cisplatin chemotherapy-induced tissue toxicity.

## 1 Introduction

Acute kidney injury (AKI) is characterized by a prompt reduction in renal function and glomerular filtration rate (GFR) together with the accumulation of waste products such as creatinine and urea in the blood. AKI can be caused by toxic and or ischemic insults caused by antibiotics, chemotherapy and shocks from serious infection or surgery (1, 2). Cisplatin, one of the most prescribed chemotherapeutic agents, is often limited in efficiency by its greatest detrimental side effect, AKI. The nephrotoxic side effect is a result of cisplatin absorption and accumulation in tubular epithelial cells and subsequent oxidative stress and inevitable regulated cell death (3, 4). Cisplatin-induced AKI comprises vascular injury, inflammation and proximal tubular injury (5–7). AKI patients have a significant risk of developing chronic kidney disease (CKD), which could possibly lead to end-stage renal disease (ESRD). ESRD current therapies involve dialysis or transplantation (1, 2). Thus, cell-based therapies have been suggested for treatment or prevention of cisplatin chemotherapy-induced tissue toxicity.

Mesenchymal stromal cells (MSCs) can be found in bone marrow, adipose tissue, periosteum, synovium, dental pulp, fetal tissues, umbilical cord tissue, placenta, amniotic fluids, and skin. These cells show fibroblast-like morphology when cultured *in vitro* and are capable of chondrogenic, adipogenic and osteogenic differentiation (8). MSCs possess distinct therapeutic properties such as being anti-inflammatory, anti-apoptotic, anti-fibrotic, anti-oxidant and pro-regenerative. These therapeutic properties of MSCs are attributed to their ability to secrete growth factors, cytokines, and various anti-oxidants (9–12). Accordingly, MSCs have been evaluated as a potential therapy for many different pathologies, including AKI (9). ATP-binding cassette member B5- positive (ABCB5+) MSCs are a non-hematopoietic cell subset in human dermis characterized by a single molecular marker (P-glycoprotein ABCB5) (13). ABCB5+ MSCs have already demonstrated therapeutic efficacy in clinical trials in chronic skin wounds and in recessive dystrophic epidermolysis bullosa (14, 15). Preclinical, ABCB5+ MSCs were able to reverse metabolic reprogramming in polycystic kidney cells (16). Based on these encouraging results, we decided to investigate the potential efficacy of these cells for treatment of cisplatin-induced kidney injury.

First, we performed a series of *in vitro* experiments to elucidate the anti-apoptotic/anti-necroptotic and immunomodulatory capacity of these cells. Since it was shown that ABCB5+ MSCs had a similar renoprotective effect compared to adipose-derived stromal cells (ASCs) in a polycystic kidney disease (PKD/MHN) model (16), we included ASCs in our *in vitro* study for comparison. Contrary to clinical-grade ABCB5+ MSCs, which were administered immediately upon thawing of cryopreserved stock, ASCs were laboratory-grade and harvested freshly from culture. Moreover, as we previously observed that the efficacy of MSCs in modulating the immune system could be compromised by cross-species incompatibility (17), we investigated the immunomodulatory effects of human ABCB5+ MSCs using both human and rat-derived immune cell co-cultures, and compared it to co-cultures involving rat bone marrow-derived MSCs (rMSCs). This inter-species study was considered particularly important, as the murine graft recipient model is a standard pre-clinical animal model before clinical translation. Discrepant outcomes of preclinical and clinical studies might be explained by species-specific dynamic interactions of immune responses and the resulting differences in modes of action.

Finally, clinical-grade ABCB5+ MSCs were administered in a therapeutic AKI model, injecting a single dose of ABCB5+ MSCs either intravenously (i.v.) or intraperitoneally (i.p.) on day 3 after cisplatin treatment. Renal damage was assessed at physiologic, metabolic, and histologic levels, as well as at gene expression levels assessing mRNA changes on day 14 post injury.

## 2 Materials and methods

### 2.1 Cell culture

Clinical-grade ABCB5+ MSCs were provided by TICEBA GmbH (Heidelberg, Germany) (18) and were thawed and either seeded directly in the experimental plate the night prior to the experiments or formulated for injection. The cells were cultured in Ham´s F-10 media supplemented with 15% stem cell media (proprietary formulation TICEBA), 1% Penicillin-Streptomycin (100,000 U/ml penicillin and 10 mg/ml streptomycin; PAN Biotech, Aidenbach, Germany) and 4% L-glutamine (4 mM; PAN Biotech).

Adipose stromal cells (ASCs) were isolated from healthy donors after having obtained informed consent. The study was approved by the Mannheim Ethics Commission II (vote numbers 2010-262 N-MA, 2009-210 N-MA, 49/05 and 48/05). ASCs were isolated using collagenase digestion (collagenase NB4, 0.15 PZ-U/ml Serva Electrophoresis GmbH, Heidelberg, Germany), seeding the obtained stromal vascular fraction in Dulbecco´s Modified Eagle Medium (DMEM) low glucose (PAN Biotech) supplemented with 10% pooled human serum from healthy AB donors (German Red Cross Blood Service Baden-Württemberg - Hessen, Mannheim, Germany, supplemented with 1% Penicillin/Streptomycin and 4% L-glutamine (both PAN Biotech). After the first passage with trypsin/EDTA (0.05% trypsin, 0.02% EDTA, PAN Biotech, ASCs were continuously cultured at a seeding density of 200 cells/cm^2^ consecutively underwent extensive characterization regarding proliferation, adipogenic and osteogenic differentiation and immune phenotyping (19).

Rodent MSCs (rMSCs) were isolated from bone marrow of male SD rats (200 g) and cultured in DMEM medium supplemented with 10% fetal bovine serum (FBS, Thermo Fisher Scientific, Waltham, USA), 1% Penicillin/Streptomycin and 4% L-glutamine (20). rMSCs underwent flow cytometric characterization as described below.

ASCs and rMSCs were cryopreserved in FBS with 10% Dimethyl sulfoxide (DMSO) (Wak-chemie Medical GmbH, Steinback, Germany).

Conditionally immortalized proximal tubular epithelial cells (ciPTECs) 14.4, stably transfected by SV40T and hTERT, were obtained from Cell4Pharma and grown at 33°C to allow proliferation according to (21) in DMEM-F12 (Thermo-Fischer, supplemented with insulin/transferrin/selenium (ITS, 5 µg/ml, Sigma Aldrich, Merck, Darmstadt, Germany) epithelial growth factor (EGF, 10 ng/ml, Sigma Aldrich), hydrocortisone (36 ng/ml, Sigma Aldrich), triiododthyronine (I3, 40 pg/ml Sigma Aldrich), 10% FBS (Thermo Fischer) and passaged using accutase (Sigma Aldrich). For maturation, ciPTECs were seeded in 96 well plates with 5,000 cells/well and transferred to 37°C for 7 days, where cells enter growth arrest and express differentiation markers.

### 2.2 MSC immunophenotyping

ABCB5+ MSCs, ASCs, and rMSCs were characterized by flow cytometry. ASCs from multiple donors were analyzed at passage 2, whereas ABCB5+ MSCs (passage 7 to 9) were analyzed immediately after thawing, (antibodies used and ABCB5+ versus ASC phenotype: see **Supplementary Table 1**; **Supplementary Figure 1**, respectively).

rMSC phenotype was ascertained using the following rat antibodies: anti-CD45-FITC (Clone OX-1; Bio-Rad, California, USA) to exclude hematopoietic cells, anti-CD90-PE (Clone OX-7; BD Biosciences, Heidelberg, Germany) and anti-CD44-APC (Clone 12K35; Lifespan Biosciences, USA) as typical MSC markers. SYTOX blue dead cell stain (1:2,000 final dilution; Thermo Fisher Scientific) was used as a viability dye. All antibodies were properly titrated. A minimum of 10,000 labeled cells were acquired with a BD FACS Canto II flow cytometer (BD Biosciences) and.fcs files were analyzed with FlowJo 10 software (FlowJo, LLC, Ashland, OR, USA).

### 2.3 Conditioned medium production

Conditioned medium (CM) was produced from naïve unprimed MSCs as increasing evidence shows that the paracrine factors secreted by MSCs (collectively termed “secretome”) are part of their therapeutic effects (22–24). For CM production of ABCB5+ MSCs, frozen cells were thawed, washed, and seeded at a density of 2.5 x 10^4^ cells/cm^2^ in ABCB5+ MSC culture medium and cultured overnight to allow cell adherence. The next day adherent ABCB5+ MSCs were washed twice with Dulbecco’s phosphate-buffered solution (DPBS) and the medium was then replaced with either serum-free medium (SFM) for ciPTECs (for ciPTEC experiments) or X-Vivo 10 (Lonza, Verviers, Belgium, for macrophage experiments). 24 h later the CM was harvested, centrifuged 2,000 x g in 4°C for 10 min and filtered with 0.22 µm filter to remove cell debris. CM from ASCs and rMSCs were produced once the cells reached 80-90% confluence as described above. As control (CTRL) medium, ciPTEC serum free medium (SFM) and X-Vivo 10 were incubated for 24 h in empty = cell-free culture flasks (37°C, 5% CO_2_) and were then processed in the same manner as CM.The CM was then stored in -80°C until further use. At least three different donors per MSC type were used.

### 2.4 Apoptosis assay

Matured ciPTECs were first treated with 15 µM cisplatin (TEVA GmbH Ulm, Germany) in ciPTEC SFM for 1 h prior to adding CM plus 15 µM cisplatin for another 23 h. ciPTECs cultured without CM (with ciPTEC SFM) in absence or presence of cisplatin served as healthy and cisplatin-treated control (CTRL), respectively. After 24 h, cisplatin-containing medium was discarded and the cells were washed once with HBSS (PAN Biotech). After cisplatin removal, fresh CMs or ciPTEC SFM were added containing 50 nM Apotracker Green (BioLegend, San Diego, USA) and Incucyte^®^ Cytotox Red Dye (12.5 nM, Sartorius, Goettingen, Germany). Apoptosis and necrosis/necroptosis was monitored for 3 days after cisplatin removal by live-cell imaging with 3h intervals (Incucyte SX5, Sartorius). Total integrated intensity of green fluorescence (green calibrated units GCU x µm^2^/image) representing apoptotic ciPTECs was normalized by cell confluence (%) and presented as relative value to cisplatin-treated ciPTEC in SFM (cisplatin CTRL). We focused on addressing apoptosis, as previous studies reported that low dose cisplatin (3-50 µM) induces apoptosis rather than necrosis/necroptosis, induced by transient high dose treatment (300 – 1000 µM) (25). Verification, however, was done using flow cytometry measuring annexin V (Annexin V FITC, 2.25 µg/ml, Biolegend with 1x Annexin V binding buffer, BD and blocking peptide as control 50 µg/ml Annexin V purified, BD final conc.) versus propidium iodide (2 µg/ml, Sigma) using staurosporine (1µM, for 4h, Abmole, Houston, USA) as positive control.

#### 2.4.1 Thiol measurement of CM

Free thiols are sulfhydryl groups (R-SH) found in peptides and proteins involved in the cellular redox state. Highly sensitive to reactive oxygen species, free thiols serve as redox switch and scavenge reactive oxygen species (ROS) (26). To predict the anti-oxidative capacity of ASC CM, we measured the concentration of free thiols (Thiol Quantification Assay kit, Abcam, Cambridge, UK). The standard and the thiol green indicator solution were prepared according to the manufacturer’s manual. 50 µL of standard solution or CM (equilibrated to room temperature and centrifuged to remove any debris) were incubated with 50 µL of thiol green indicator solution for 10 min in the dark. The fluorescence was read using a microplate reader (Infinite M200, Tecan, Männedorf, Switzerland) at ex/em: 490/520 nm.

#### 2.4.2 Treatment with apoptosis and necrosis inhibitors

To understand the mechanisms by which ASC CM inhibits apoptosis, we applied the following inhibitors within the live cell apoptosis assay: necrostatin-1 (Nec-1, inhibitor of necroptosis and ATP-competitive inhibitor of Receptor-interacting serine/threonine-protein kinase 1 (RIPK1), 40 µM, Biomol), sphingosine kinase inhibitor (SKI-II, 5 µM, Biomol), mithramycin A (Mit-A, 40 nM, Biomol), N-acetylcysteine (NAC, ROS inhibitor, 7.5 µM, Sigma), and anti- human-stanniocalcin-1 (anti-STC-1, 2 µg/ml, R&D Systems). All inhibitors were properly titrated (balancing inhibition versus target cell toxicity) and added together with the CM after the 1 h incubation with cisplatin only, and with the fresh CM after 24 h of cisplatin treatment. Three independent experiments were performed using a pool of CM, pooled from N=3 ABCB5+/ASC donors.

### 2.5 Suppression of peripheral blood mononuclear cells proliferation

#### 2.5.1 Isolation of human- and rat-derived peripheral blood mononuclear cells

Human-derived peripheral blood mononuclear cells (hPBMCs) were isolated from leukapheresis samples from healthy donors provided by the German Red Cross Blood Service Baden-Württemberg - Hessen, Institute Mannheim, after obtaining informed consent. Rat PBMCs (rPBMCs) were isolated from freshly collected blood of SD rats. h/rPBMC isolation was performed following a standard Ficoll-Paque™ density gradient isolation (GE Healthcare Bio-science AB, Uppsala, Sweden).

#### 2.5.2 h/rPBMC proliferation assay

For testing h/rPBMC proliferation, allogeneic and xenogeneic co-cultures were performed (17). The setting of the allo-cocultures were: 1) hMSCs (ABCB5+ MSCs/ASCs) + hPBMCs, 2) rMSCs + rPBMCs; while xeno-cocultures were: 3) hMSCs (ABCB5+ MSCs/ASCs) and rPBMCs, 4) rMSCs + hPBMCs.

2x10^4^ MSCs from different donors were seeded a day before PBMC co-culture to allow attachment in Roswell Park Memorial Institute (RPMI) 1640 media (Lonza) supplemented with 10% FBS (for hPBMCs) or 10% heat inactivated FBS (for rPBMCs) according to the experiment, 1% Penicillin/Streptomycin and 4% L-glutamine. hPBMCs or rPBMCs were stained with the proliferation dye Cytotell Green (20 µM/ml, ATT Bioquest, Sunnyvale, USA). 1x10^5^ h- or rPBMCs were seeded on top of the MSCs in a direct co-culture (ratio 1:5 MSCs: PBMCs) and stimulated with either phytohemagglutinin (PHA,1.25 µg/ml Merck Millipore, Darmstadt, Germany), for hPBMCs or concanavalin A (ConA, 4µg/ml, Sigma Aldrich) for rPBMCs. Interleukin-2 (rhIL-2, 1 µg/ml, Promocell GmbH, Heidelberg, Germany) for hPBMCs and β-mercaptoethanol (50 µM, Sigma-Aldrich) for rPBMCs were added to the culture. Co-culture ran for 3 days for ConA-stimulated co-cultures and 5 days for PHA-stimulated co-cultures. Supernatants (SN) of the co-cultures were harvested and frozen at -80° C. PBMCs were collected in FACS tubes, stained with the dead cell stains Sytox Red (final concentration 1.25 nM/ml) or Sytox Blue (final concentration 0.5 µM/ml, both Invitrogen Life Technologies, Oregon USA). PBMC proliferation was assessed via Cytotell green dye dilution using a BD FACS Canto II flow cytometer (BD Biosciences). The.fcs files were analyzed using the proliferation tool from FlowJo™ 7 Software v10.8 Software (BD Life Sciences), which allowed us to monitor cell division over time due to its uniform distribution among daughter cells in each division. The Division index (DI), i.e., the average number of cell divisions that a cell in the original population had undergone, was chosen to define PBMC proliferation.

#### 2.5.3 Measurement of kynurenine and nitrite in the conditioned media of the cocultures

Kynurenine (Kyn) and nitrite concentrations were measured in co-culture SN of 3-day co-cultures under stimulated or un- stimulated conditions.

For Kyn, 100 µl of standard (50 mM L-kynurenine, Santa Cruz Biotechnology, Heidelberg, Germany) or sample was added to 50 µl of 30% trichloroacetic acid (Carl Roth GmbH, Karlsruhe, Germany). The samples were incubated for 30 minutes at 50°C, then centrifuged (10 minutes at 4,000 x g) and then 75 µl of the supernatant was mixed with 75 µl 2% 4-(Dimethylamino) benzaldehyde (Santa Cruz Biotechnology). After 15 minutes at RT, the optical density (OD) was measured (TECAN infinite M200PRO, Tecan Deutschland GmbH, Crailsheim, Germany) at 492 nm emission. Standard curves were constructed using GraphPad Prism 9.1.0 Software (GraphPad Software, California, USA).

For nitrite measurements, 150 µl of standard sodium nitrite (0.1 µmol/ml sodium nitrite dissolved in RPMI, Applichem, Darmstadt, Germany) or samples were diluted in RPMI 1640 media, mixed with 50µl of sulfanilamide (AppliChem) and incubated for 2 minutes. 50 µl of N-(1-Napthyl)-ethylendiamine dihydrochloride (naphtylamine) (Carl Roth GmbH) was added before another incubation for 30 minutes at RT in the dark. The OD was measured using 542 nm emission/620 nm. Standard curves were constructed using GraphPad Prism 9.1.0 Software and the limit of detection was calculated.

### 2.6 Macrophage polarization assay

#### 2.6.1 CM treatment of human macrophages

MSC-CM was used to treat human macrophages (hMac) derived from primary monocytes. Human monocytes were isolated from buffy coats provided by the German Red Cross Blood Service Baden-Württemberg – Hessen. Buffy coats from eight healthy blood donors were used after having obtained informed consent. Monocyte isolation was performed by positive selection using anti-human CD14 Microbeads and magnetic activated cell sorting (MACS) (Miltenyi Biotech, Bergisch Gladbach, Germany) according to the manufacturer’s instruction.

10^5^ monocytes/well were seeded in 96-well plates in CM obtained from ABCB5+ MSCs, ASCs and rMSCs (each cell type from 3 different donors) or X-Vivo CTRL medium supplemented with 10 ng/ml human macrophage colony-stimulating factor (MCSF) (Peprotech, Cranbury, USA). Monocytes were then cultured for 6 days without media change until they matured into macrophages.

#### 2.6.2 Rat monocyte isolation and CM treatment

Rat monocytes were isolated from SD rats’ femurs (untreated control animals from other projects, no additional animal license needed for this project). Rat bone marrow was flushed out after cutting both end of the femurs. Isolated bone marrow was treated with red blood cell lysis buffer (1.55 mM NH_4_Cl, 0.1 M NH_4_HCO_3_, 1 mM EDTA) for 10 min, washed twice with PBS-EDTA (2 mM; Applichem) and filtered through 70 µm nylon filter. Monocyte isolation was then performed by positive selection against CD11b+ cells using PE-conjugated anti-rat CD11b/c antibody (clone REA325) and anti-PE Microbeads (both from Miltenyi Biotech) consecutively. Cell separation was done as per the manufacturer’s manual. 10^5^ monocytes/well were seeded in 96-well plates in CM or normal X-Vivo 10 medium, supplemented with 25 ng/ml murine MCSF (Peprotech) and cultured for 6 days without media change for maturation into macrophages (rMac).

#### 2.6.3 Phagocytosis assay

Phagocytosis capacity of hMac and rMac was assessed at the end of 6 d culture in CM by adding 5 µg/ml PHrodo-conjugated *E. coli* bioparticles (Sartorius, Carlsbad, USA) into each well. The phagocytosis was imaged by live-cell imaging device (Incucyte SX5, Sartorius, Goettingen, Germany) every 15 min for 6 h. Total integrated intensity of green fluorescence (GCU x µm^2^/image) representing the cells phagocytosing the bioparticles was normalized by cell confluence (%, measured with Incucyte SX5) by calculating percentage of total phase object area, and presented as fold change to control group (CTRL).

#### 2.6.4 Lipopolysaccharide-stimulated TNF-α release

Lipopolysaccharide (LPS from *E. coli*, 100 ng/ml, Sigma Aldrich) stimulation of hMac and rMac to trigger TNF-α secretion was done after 6 d culture in CM. LPS was added without media change. 24 h after LPS stimulation, culture SN was harvested, centrifuged at 420 x g for 10 min and stored in -80°C for further use.

TNF-α levels in hMac and rMac SN were assessed using human TNF-α DuoSet and rat TNF-α DuoSet ELISA (both R&D Systems, Minneapolis, USA), respectively, as per manufacturer’s instructions. The level of TNF-α was normalized to cell confluence (%, measured with a Incucyte SX5) and presented as relative value to LPS-stimulated CTRL.

#### 2.6.5 Inhibition of MSC secreted factors acting on macrophages

For inhibition of MSC-secreted factors, the CM from 3 different donors of each MSC type were pooled. After isolation, hMac and rMac seeded in the presence and absence of CM were treated with inhibitors in MSC CM for the entire maturation process (6 days). The inhibitors used were: anti-Interleukin-6 (IL-6) receptor-α antibody Sarilumab 0.4 µg/ml (R&D Systems) as IL-6 inhibitor, GW788388 2.5 µM as transforming growth factor- β1 (TGF-β1) inhibitor, L-161,982 20 µM as prostaglandin E_2_ (PGE-2) inhibitor and bindarit 18.75 µM (all three compounds from Cayman Chemical, Michigan, USA) as monocyte chemoattractant protein-1 (MCP-1) inhibitor. The effect of these inhibitors on hMac and rMac was assessed using phagocytosis assay.

### 2.7 Animal model of cisplatin-induced AKI

The animal study was conducted in accordance with the German Animal Protection Law and approved by the local authority (Regierungspräsidium Karlsruhe, Germany) in agreement with EU guideline 2010/63/EU.

Male Sprague Dawley (SD) rats (200g) (Janvier Labs, Le Genest Saint Isle, France) were provided with a pelleted standard rat chow diet (rat/mouse maintenance V1534-0001, Ssniff, Soest, Germany) and free access to water in a temperature- and humidity-controlled facility (22°C, 55+/-5% relative humidity) with 12 h light/12 h dark cycle.

#### 2.7.1 Development of cisplatin-induced nephropathy model in SD rats and assessment of the therapeutic potential of human ABCB5+ MSCs

Kidney failure was induced in male SD rats (~350 g) by a single i.p. administration under anesthesia (xylazine 2%, 5 mg/kg BW and ketamine 10%, 100 mg/kg BW) of cisplatin (1 mg/ml solution) at a dose of 7 mg/kg body weight (BW) (day 0). 50 animals were involved in the experiment, including N=3 non-cisplatin-treated control animals. After cisplatin administration, rats were randomly allocated to different groups according to the treatment on day 3:

1) cisplatin control (CTRL) N=19;
2) cisplatin + vehicle CTRL N=4: 0.5 ml of fresh HRG solution (49.5% of 5% human serum albumin (HSA), 49.5% Lactated Ringer´s solution, 1% of 40% glucose);
3) cisplatin + i.v. ABCB5+ MSCs N=12: 2x10^6^ ABCB5+ MSCs i.v. administrated in HRG;
4) cisplatin + i.p. ABCB5+ MSCs N=12: 2x10^6^ ABCB5+ MSCs i.p. administrated in HRG.

The animals’ state of health was checked 3 days before the start of the experiment (baseline), and 2, 7 and 14 days after cisplatin/saline administration. At any timepoint, animals were placed into metabolic cages for 16 hours to collect urine and to record any changes in BW, diuresis, food, and water intake. Subsequently, blood samples were collected via the ophthalmic venous plexus under anesthesia (xylazine and ketamine). Further, transcutaneous renal function (see below) was assessed. Plasma and urine chemical parameters were determined using the Cobas c311 analyzer (Roche Diagnostics GmbH, Mannheim, Germany). Urinary albumin was determined by ELISA assay and osmolarity was analyzed using an osmometer (2020 Multi-Sample Osmometer, Advanced Instruments Inc., Norwood, MA).

Animals were sacrificed on day 15 by perfusion (0.9% saline heparin 5 IU/mL pH=7 for 3 min at 280 mbar, paraformaldehyde (PFA) 4% for 3 min at 230 mbar) under anesthesia (xylazine and ketamine). Fresh left kidney was collected before perfusion and immediately snap-frozen in liquid nitrogen for subsequent mRNA extraction. Other organs (right kidney, spleen, liver, pancreas, intestine, heart, lungs) were collected after perfusion and processed for subsequent histology analyses.

#### 2.7.2 Transcutaneous assessment of renal function

Renal function was measured using a transdermal device (MediBeacon GmbH, Mannheim, Germany), the optical part of which consists of two light-emitting diodes and a photodiode detecting the fluorescent light (excitation: 706 nm, emission: 790 nm). The animals received a dose of 15 mg/100 g body weight of ABZWCY-HPβCD dye [stock solution 160 mg/ml (Cyanagen, Bologna, Italy) in Deltajonin (Deltamedica, Reutlingen, Germany)] via tail-vein injection, and its emitted fluorescent signal, which represents its excretion curve, was recorded by the device (27). The measurement was performed for at least 2 hours, during which the animal was completely awake and freely moving. Half-life excretion of the administered ABZWCY-HPβCD was calculated based on a 3-compartment model using the open source freely-available software ‘GFR measure’ (https://www.mathworks.com/products/compiler/matlab-runtime.html) (28).

#### 2.7.3 Histology collection, staining, analysis and microscopy

Organs were collected after perfusion and stored in 4% PFA for 24 h. Tissues were fixed and embedded in paraffin, cut (3 μm) and stained with hematoxylin and eosin (H&E). H&E slices were analyzed to detect morphological changes induced by cisplatin. Full organ images were acquired using Axio Scan.Z1 microscope (ZEISS, Oberkochen, Germany).

To quantify the cisplatin-induced pathology, image analysis was performed using the open-source quantitative pathology software QuPath (29). A pixel classifier threshold detected dilated tubuli and hyaline casts (**Supplementary Figure 3**). To quantify the inflammatory cells, QuPath was used to train a Random Trees object classifier on regions of interest (ROIs) from adrenal cortex, in a two-step process. First, all images are normalized via estimating the stain vectors and performing color deconvolution (30). Next, proper manual annotations were performed to allow for training of the object classifier. An automatic cell detection algorithm, to determine total cell count in the selected area, followed this. Using the QuPath-based cell classification method, inflammatory cells were discriminated based on stain intensity and cellular morphology.

#### 2.7.4 RNAseq gene expression profiling and bioinformatics evaluation

To extract total RNA from frozen left kidney samples obtained from sacrificed animals, the RNeasy mini kit (Qiagen, Hilden, Germany) was used following the manufacturer’s instructions. The mRNA purity and integrity were tested by capillary electrophoresis (Agilent 2100 bioanalyzer, Agilent, Santa Clara, California, USA) and high quality was confirmed. The isolated RNA was used for gene expression analysis.

Next generation sequencing gene expression profiling was performed with RNA sequencing (RNAseq) technology (BGI Tech Solutions Co., Hong Kong, China) using the BGISEQ-500 method. Quality control of raw sequencing reads was performed using FastQC (Babraham Bioinformatics). Low-quality reads were removed using preprocessReads from the SystemPipeR (31) package in R (parameter: cutoff = 20, low_quality_rate = 0.4, batchsize = 100,000). The resulting reads were aligned to rat genome version rn6 from UCSC using tophat2 (32). Reads counting was performed with the summarizeOverlaps function from the GenomicsAlignments (33) package in R. EdgeR (34) was used to perform a differential expression analysis. A false positive rate of α = 0.05 with FDR correction was taken as the level of significance. Gene Set Enrichment Analysis (GSEA) was performed by using the Kyoto Encyclopedia of Genes and Genomes (KEGG) database (version September 2018) (https://www.ncbi.nlm.nih.gov/geo/query/acc.cgi?acc=GSE223675).

### 2.8 Statistical analysis

All statistical calculations were performed using GraphPad Prism 9.1.0 software. The statistical test run for each data set is indicated in the figure legend. Mean ± standard deviation are depicted. N indicates biological replicates for each cell type or animal, while n refers to technical replicates. Statistical significance is indicated by *p<0.5, **p<0.01, ***p<0.001, ****p<0.0001.

## 3 Results

### 3.1 Clinical-grade ABCB5+ MSC CM decreased cisplatin-induced apoptosis of ciPTECs

**Figure 1:**
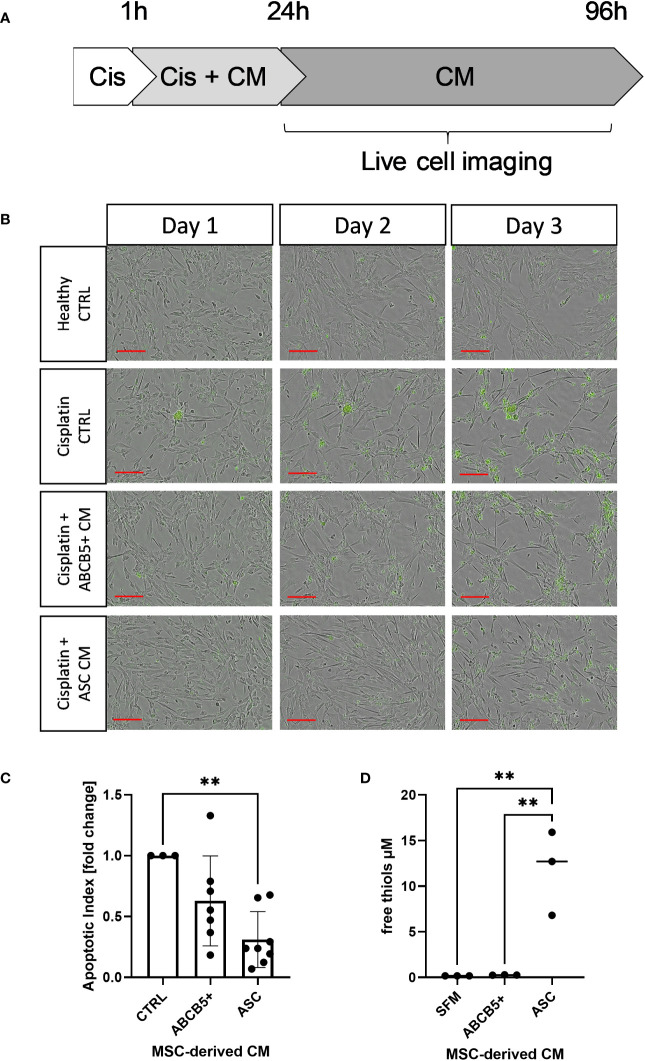
Conditioned medium from ABCB5+ MSCs and ASCs decreases cisplatin-induced apoptosis of ciPTECs. **(A)** CiPTECs were treated with cisplatin for 1 h and then further 23 h in the absence and presence of MSC conditioned medium (CM). After 24 h, cisplatin-containing medium was replaced with fresh CM or CTRL medium without cisplatin and in the presence of Apotracker. Apoptosis of ciPTECs was monitored by live cell imaging for 3 days. **(B)** Representative images of ciPTEC day 1, 2 and 3 after cisplatin treatment started. Green: apoptotic cells. Scale bars: 200 µm. **(C)** Quantification of ciPTEC apoptosis on day 3. The bars indicate mean fold change relative to cisplatin-CTRL; **(D)** Quantification of free thiol content in MSC-derived CM. One-Way ANOVA with Tukey’s multiple comparisons test; **p<0.01, cisplatin CTRL N=1, ABCB5+ CM N= 3, ASC CM N= 4, ciPTEC n=3.

First, the anti-apoptotic capacity of ABCB5+ MSCs, specifically the CM of naïve ABCB5+ MSCs, was assessed in an *in vitro* cisplatin-induced renal injury model and compared to CM from ASCs, which have shown efficacy in PKD/MHM rats (16). For this experiment, matured ciPTECs were used as they express organic cation transporter-2, which renders them sensitive to cisplatin injury (35, 36). Cisplatin-induced ciPTEC apoptosis progressed over time as marked by the increase of Apotracker-labeled cells (**Figures 1A, B**). ciPTECs treated with ABCB5+ MSC CM showed on average 37% less apoptosis compared to cisplatin CTRL (fold change: 0.63 ± 0.37, **Figure 1C**). However, the apoptosis reduction was not statistically significant due to one donor of ABCB5+ that stimulated apoptosis. ASC CM, serving as positive control, significantly suppressed ciPTEC apoptosis on average by 70% as compared to cisplatin control (fold change: 0.31 ± 0.23). We noted that ciPTECs treated with ASC CM showed higher confluence than the control and ABCB5+ MSC CM-treated groups (**Figure 1B**). Thus, we normalized data for cell confluence. These data indicate that CM derived from naïve clinical-grade ABCB5+ MSCs are able to attenuate ciPTEC apoptosis.

**Figure 2:**
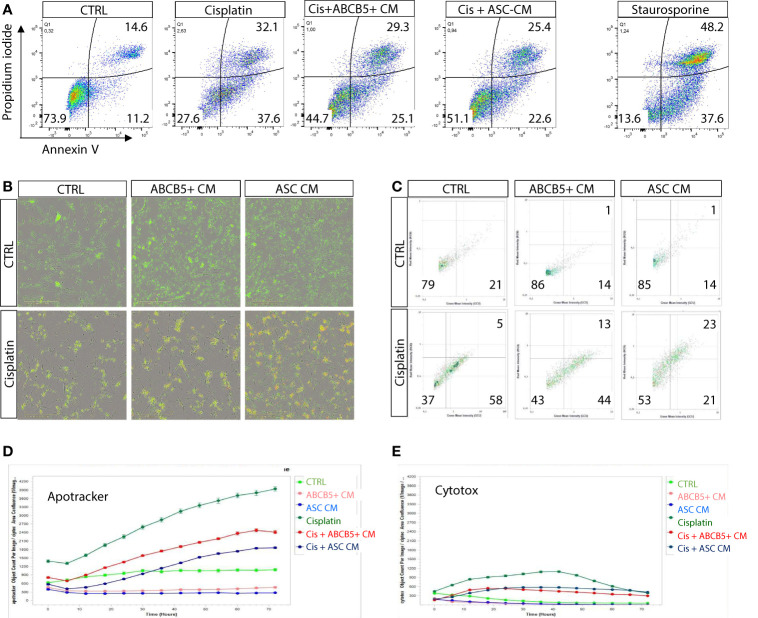
Cisplatin induces predominantly apoptosis rather than necrosis/necroptosis, ameliorated by MSC conditioned medium. **(A)** Representative dot plots of ciPTEC analyzed by flow cytometry for early and late apoptotic cells by staining for phosphatidylserine with Annexin V and with the DNA binding dye propidium iodide (PI). Cells were classified as early apoptotic with Annexin V-only positive staining (lower right quadrant), and late apoptotic (or dead) cells with Annexin V and PI positive staining (upper right quadrant). H_2_O_2_ was used as positive control. **(B)** Representative images of live-cell imaging experiments of ciPTEC stained with Apotracker (green) and sytotox (red) and **(C)** representative dot plots of Apoptracker vs Cytotox as shown for flow cytometry data. Quantification of Apotracker positive **(D)** or Cytotox-positive objects **(E)** normalized against confluence. One representative experiment is shown.

Previous data indicated induction of either apoptosis or necrosis/necroptosis by cisplatin, depending on the cisplatin dose and incubation period (25). Using flow cytometry we verified that MSC CM affected mainly cisplatin-induced apoptosis, rather than necrosis (**Figure 2A**). Similar data were observed in the live-cell imaging analyses, using Apotracker and Cytotox dyes (**Figures 2B–E**).

**Figure 3:**
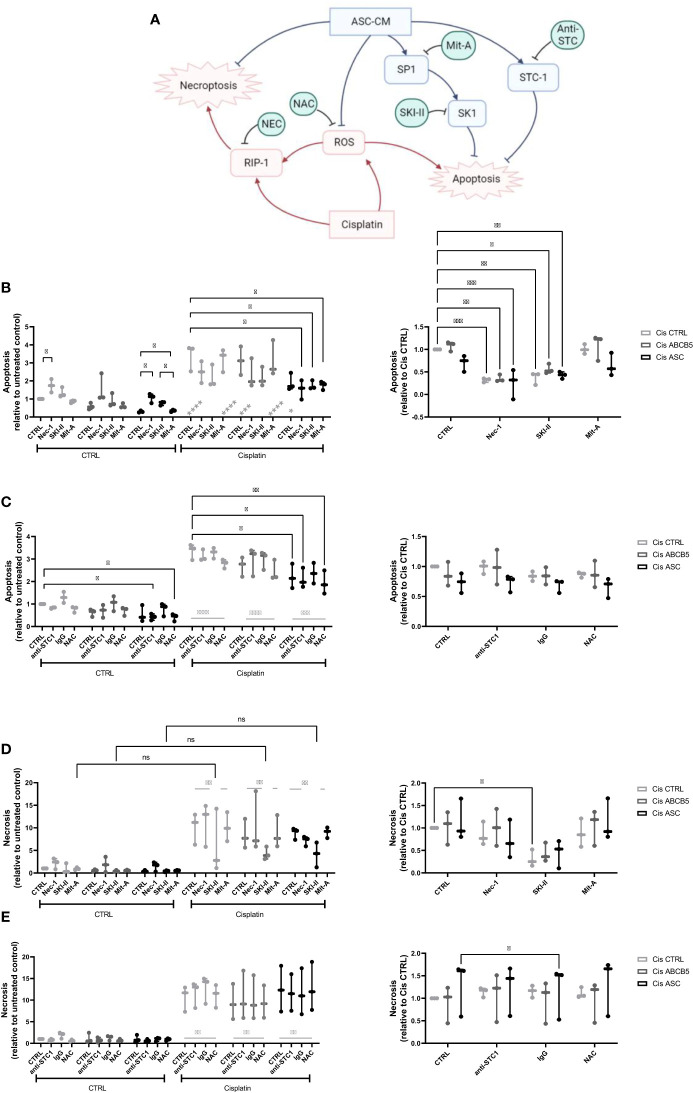
Mechanistic studies on pathways affected by cisplatin and MSC conditioned medium. **(A)** Overview over pathways potentially affected by cisplatin and MSC conditioned medium treatment and addressed by the respective inhibitors, Nec-1, SKI-II, Mit-A, anti-STC1, and NAC. **(B, C)** Apoptosis and **(D, E)** necrosis of ciPTEC treated with cisplatin and MSC CM and the respective inhibitors Nec-1, SKI-II, Mit-A **(A, D)**, anti-STC1, IgG as control, and NAC **(B, E)**. The left panel shows the data relative to the untreated control, the right relative to the Cis control. Two-Way ANOVA with Tukey’s multiple comparisons test; *p<0.05, **p<0.01, ***p<0.001, ****p<0.0001. Nec-1 - necrostatin, SKI-II - sphingosine kinase inhibitor, Mit-A – mithramycin-A, anti-STC-1 – anti-stanniocalcin-1, NAC - N-acetylcysteine. ns, not significant.

Within a previous study, we observed that ASC secretome attenuated cisplatin-induced cell toxicity and promoted migration of ciPTECs ameliorating apoptosis, expression of nephrotocicity genes, DNA damage, and oxidative stress (Rendra et al. submitted). This effect was independent of extracellular vesicles, but related to the free thiol content within the ASC CM. In this study, we reproduced the findings that ASC CM contains free thiols at varying levels (**Figure 1D**). Interestingly, ABCB5+ CM, although it inhibited apoptosis to 37%, contained no detectable free thiols. To further address potential mechanisms, we applied a variety of inhibitors for pathways described to be modified by MSCs, MSC-derived secretome/conditioned medium or their extracellular vesicles (EVs) (37–39): necrostatin-1 (Nec-1, RIP-1 and necroptosis inhibitor); sphingosine kinase inhibitor (SKI-II), mithramycin-A (Mit-A, SP1 inhibitor), anti-stanniocalcin-1 (anti-STC, and IgG as isotype control), and finally N acetylcysteine as ROS scavenger (**Figure 3A**). First, addressing apoptosis, Nec-1 induced apoptosis even in the CTRL, non-cisplatin condition, significantly for CTRL and ASC. In the CTRL setting, Nec-1, SKI-II and Mit-A reduced the ASC CM anti-apoptotic activity significantly, for ABCB5+ CM this effect was not significant (**Figure 3B**). Upon cisplatin challenge, NEC-1 and SKI-II appeared to lower apoptosis slightly also when CM was added. The protective effect of ASC CM was obvious and significantly different to Cis CTRL, when adding Nec-1, SKI-II and Mit-A – when data were normalized against the healthy CTRL. When calculated against the Cis CTRL, Nec-1 and SKI-II led to a significant reduction of apoptosis, irrespective of whether CM was added or not. Upon use of Mit-A, no significant effects of ABCB5+ or ASC CM was calculable. Regarding anti-stanniocalcin-1 and NAC, both anti-stanniocalcin-1 and NAC boosted the anti-apoptotic activity of ASC CM in the healthy CTRL condition. Upon cisplatin challenge, this effect was significant against the Cis CTRL, but not the respective Cis inhibitor conditions (**Figure 3C**). ABCB5+ CM resulted in no significant differences. When focusing on the low-level necrosis, the SKI-II inhibitor resulted in an inhibition of cisplatin-induced necrosis, but irrespective of MSC CM (**Figure 3D**), when calculated against the healthy CTRL. Calculated against the Cis CTRL, SKI-II reduced necrosis significantly in the CTRL setting, yet effects were not significant in the presence of MSC CM. Anti-STC-1 and NAC had no significant effect on cisplatin-induced necrosis, irrespective of MSC CM (**Figure 3E**).

In summary, these data suggest that ASC and ABCB5+ secretome has profound activity protecting from cisplatin-induced nephrotoxicity, especially apoptosis, by mainly scavenging ROS (**Figure 3A**).

### 3.2 Clinical-grade ABCB5+ MSCs and rMSC inhibited proliferation of human or rat PBMCs, while ASCs showed xenogeneic functional incompatibility

**Figure 4:**
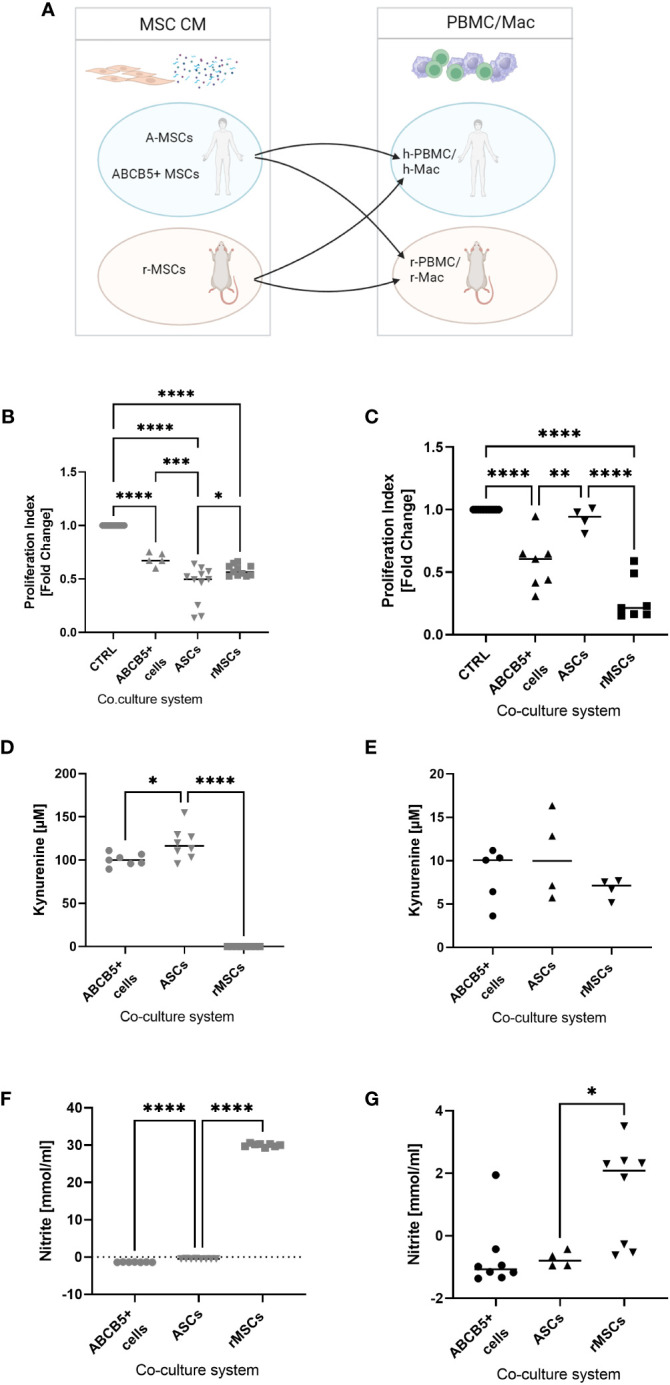
ABCB5+ cells are able to decrease PBMC proliferation in both human and rat. **(A)** Experimental layout; image created with BioRender. Proliferation index of stimulated **(B)** hPBMCs and **(C)** rPBMCs cultured alone or with MSCs. hPBMC cocultures: CTRL N=11, ABCB5+ cells N=5, ASC and rMSC N=11; rPBMC cocultures: CTRL N=11, ABCB5+ cells N=7, ASC N=4, rMSC N=7. Measurement of kynurenine and nitrite in the conditioned media of **(D, F)** MSCs-hPBMC and **(E, G)** MSCs-rPBMC cocultures. Kynurenine measurement for hPBMC cocultures: ABCB5+ cells N=7, ASC and rMSC N=8; for rPBMC cocultures: ABCB5+ cells N=5, ASC and rMSC N=4. Nitrite measurement for hPBMC cocultures: ABCB5+ cells N=7, ASC and rMSC N=8; for rPBMC cocultures: ABCB5+ cells N=8, ASC N=4, rMSC N=8. One-Way ANOVA with Tukey’s multiple comparisons test; *p<0.05, **p<0.01, ***p<0.001, ****p<0.0001.

Next, we investigated the inhibitory capacity of ABCB5+ MSCs on PBMC proliferation given that the immune system plays an important role in cisplatin-induced AKI. Having previously shown that there is xenogeneic incompatibility between human and rat MSCs : PBMCs, respectively (17),, we asked whether human ABCB5+ MSCs may show similar features. Thus, we compared the immunomodulatory capacity of human MSCs (ABCB5+ MSCs or ASCs) on human or rat PBMCs to rMSCs (**Figure 4A**). All MSCs were able to suppress hPBMC proliferation, with the highest inhibitory capacity elicited by ASCs as indicated by the proliferation index (**Figure 4B**) (fold change: 0.44 ± 0.18). On the other hand, ASCs, as shown before, could not suppress rPBMC proliferation (fold change: 0.92 ± 0.09). In contrast, both clinical-grade ABCB5+ MSCs (fold change: 0.57 ± 0.21) and rMSCs (fold change: 0.28 ± 0.18) inhibited rPBMCs proliferation (**Figure 4C**).

To unravel the underlying mechanism that causes this inter-species compatibility/incompatibility, we investigated the role of indoleamine 2,3-dioxygenase (IDO) and nitric oxide (NO), by measuring the concentration of kynurenine, a byproduct of IDO activity, and of nitrite in co-culture supernatants (17). The highest concentration of kynurenine was detected in hPBMCs co-cultured with ASCs (119.2 ± 18.25 µM), which could be associated with the strong inhibition on hPBMCs, followed by ABCB5+ MSCs (100.4 ± 7.22 µM), but none was detected in co-cultures with rMSCs (**Figure 4D**). However, nitrite concentration was found only in hPBMCs co-cultured with rMSCs (**Figure 4F**) (30.00 ± 0.43 mmol/ml). Compared to hPBMCs, kynurenine concentrations in MSCs-rPBMCs co-cultures were notably lower (8.32 ± 3.19, 10.51 ± 4.96, 6.76 ± 1.15 mmol/ml for ABCB5+ MSCs, ASCs and rMSCs, respectively) (**Figure 4E**), although the trend remained similar to the hPBMC counterparts. Nitrite concentrations in MSC-rPBMC co-culture were low and detected only in the rMSC-rPBMC system (1.37 ± 1.60 mmol/ml) (**Figure 4G**). In summary, while ASCs employ IDO to inhibit PBMC proliferation in human, and rMSCs use NO to exert immunomodulation in both species, human ABCB5+ MSCs inhibit both human and rat PBMCs, however identification of the responsible mediator requires further investigation.

### 3.3 Clinical-grade ABCB5+ MSC CM modulates hMac and rMac function

**Figure 5:**
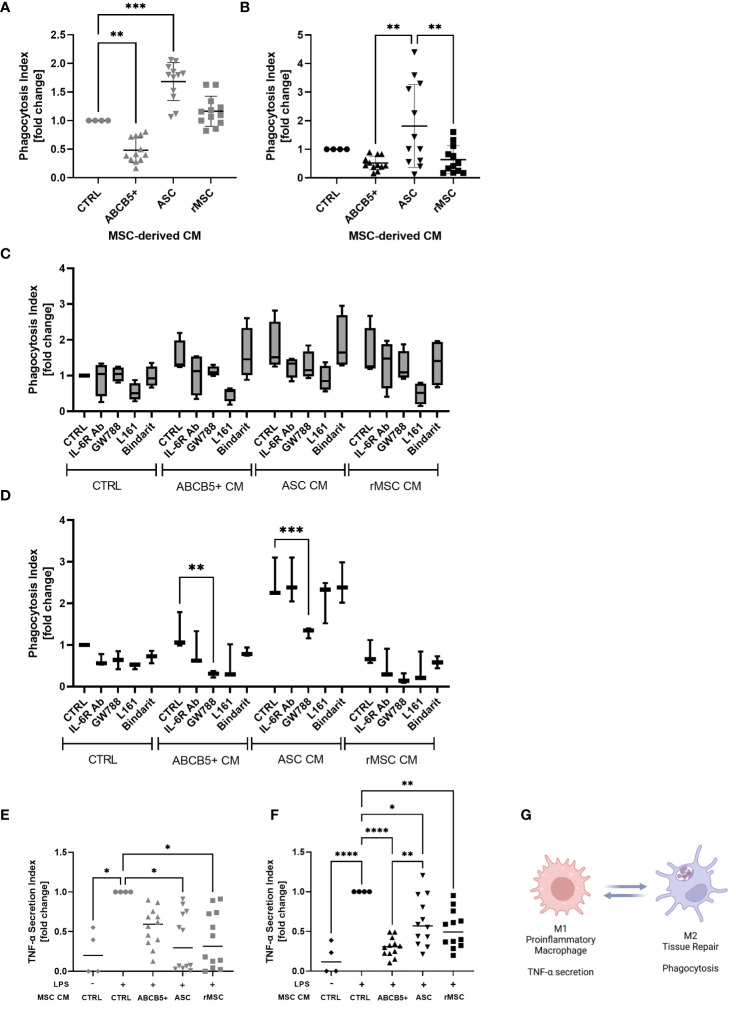
ABCB5+ cells-derived CM shows modest effect on macrophage phagocytosis but suppresses LPS-induced TNF-α secretion in both species. Experiment al layout shown in **Figure 4A**. **(A-D)** Phagocytosis capacity of **(A)** hMac and **(B)** rMac after CM treatment. hMac N= 4 and rMac N=4, ABCB5+, ASC and rMSC CM N=3. Phagocytosis of hMac **(C)** and rMac **(D)** after inhibition of MSC factors in the presence and absence of CM (pooled from N=3 MSC). hMac N=4; rMac N=3. TNF-α secretion of **(E)** hMac and **(F)** rMac after 24 h LPS stimulation (100 ng/ml) in the presence and absence of CM. hMac N= 4 and rMac N=4, ABCB5+, ASC and rMSC CM N=3. **(G)** Simplified diagram depicting M1 and M2 characteristics (Image was created using BioRender). Error bars show maximum and minimum value relative to respective CTRL; One-Way ANOVA **(A-D)** and Two-Way ANOVA **(F, G)** with Tukey’s multiple comparisons test; *p<0.05, **p<0.01, ***p<0.001, ****p<0.0001.

Macrophages are an important player in the progression of AKI as they can either exaggerate inflammation or support tissue regeneration of injured kidney (40, 41). Supporting previous studies by Giri et al., who showed that naïve MSC CM induced peritoneal macrophage M2 polarization (42), CM from ASCs significantly increased the phagocytosis rate of hMac (fold change: 1.6 ± 0.3, **Figure 5A**). rMSCs showed no significant effect (fold change: 1.2 ± 0.2), while ABCB5+ MSC CM, significantly reduced the phagocytotic index compared to control (fold change: 0.5 ± 0.2). A different pattern was observed on rMac, where none of the examined CMs significantly altered the control rMac phagocytosis rate. Human ASC CM-treated rMac nevertheless showed a tendency toward increased phagocytosis. rMSC CM- and human ABCB5+ MSC CM-treated rMacs exhibited a tendency toward decreased phagocytosis rates compared to controls (fold change: 1.8 ± 1.4, 0.6 ± 0.5 and 0.5 ± 0.2, respectively) (**Figure 5B**). To account for differing cell numbers, all data were normalized to confluence; representative light microscopy images of hMAC and rMAC cultures treated with CM from ABCB5+, ASC and rMSC are provided in **Supplementary Figure 2**.

To identify any involved factors, we inhibited some known M2-polarizing factors, IL-6, TGF-β1, PGE-2 and MCP-1 using IL-6R blocking antibody, GW788, L161 and Bindarit, respectively, concomitantly with CM treatment. In hMac, all the inhibitors seemed to have some effects on phagocytosis of CM-treated macrophages, with the PGE-2 inhibitor, L161, to be the most potent inhibitor, regardless of the CM source (**Figure 5C**). However because CTRL medium showed the same inhibition pattern this suggests that the inhibited factors were secreted by macrophages in an autocrine fashion (**Figure 5C**). In contrast, in rMac cultures the inhibitor of TGF-β1, GW788, significantly reduced rMac phagocytosis selectively in the group treated with human MSC CM, i.e. ABCB5+ MSC CM and ASC CM, with phagocytosis reduction rates of 76% and 48%, respectively (**Figure 5D**). While none of the tested candidates provided an explanation for the observed differences between ABCB5+ CM and ASC CM effects on rates of macrophage phagocytosis, our results indicate that human MSC-secreted TGF-β1 is responsible, at least in part, for maintaining M2-associated macrophage phagocytotic function in rats.

Further, the ability of CM-treated macrophages in secreting TNF-α upon LPS stimulation was evaluated as a surrogate for M1/M2 polarization following CM treatment. TNF-α plays a central role in the host response to injury and inflammation (43). All LPS-stimulated hMac cultures treated with ASC CM, rMSC CM or ABCB5+ MSC CM exhibited reduced TNF-α secretion versus the CM-untreated LPS-stimulated controls (fold change of ASC CM: 0.38 ± 0.36, rMSC CM: 0.4 ± 0.35, ABCB5+ MSC CM: 0.56 ± 0.24) (**Figure 5E**). Similarly, in rMac cultures, all MSC CMs tested lowered LPS-mediated TNF-α secretion (fold change of ASC CM: 0.62 ± 0.30, rMSC CM: 0.53 ± 0.24, ABCB5+ MSC CM: 0.30 ± 0.12) (**Figure 5F**). In aggregate, our data suggest that in the herein used model systems, both ABCB5+ MSC CM and ASC CM inhibit LPS-driven macrophage TNF-α secretion but show variable effects on the rates of M2 macrophage-associated phagocytotic activity (**Figure 5G**).

### 3.4 Xenogeneic human ABCB5+ MSC grafting in a rat model of cisplatin-induced nephrotoxicity shows no effect on recipient biochemical parameters, body weight, diuresis, food and water intake, renal function and renal morphology

**Figure 6:**
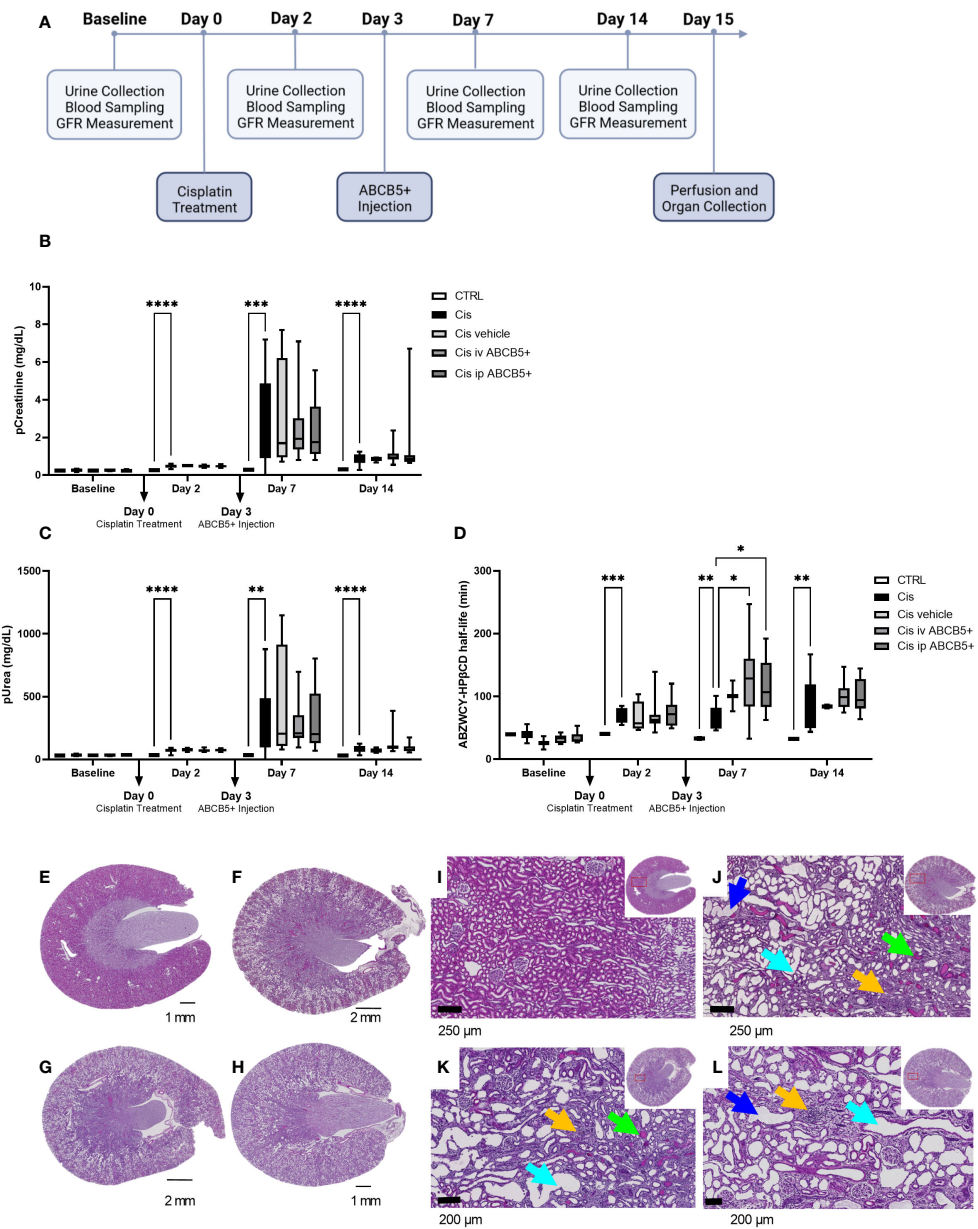
ABCB5+ MSCs do not ameliorate renal injury in rats pretreated with cisplatin. **(A)** The timeline of ABCB5+ treatment on rats pretreated with cisplatin. **(B)** Creatinine and **(C)** urea level in rats’s plasma and **(D)** ABZWCY-HPβCD half-life on the day before and 2, 7, 14 days after cisplatin treatment with and without ABCB5+ injection. The bars indicate mean value with standard deviation. For pCreatinine and pUrea: healthy CTRL N=3, cisplatin CTRL N=19, cisplatin vehicle CTRL N= 4, cisplatin i.v. ABCB5+ N=12, cisplatin i.p. ABCB5+ N=12; for ABZWCY-HPβCD half-life: healthy CTRL N=3, cisplatin CTRL N=13, cisplatin vehicle CTRL N= 4, cisplatin i.v. ABCB5+ N=11, cisplatin i.p. ABCB5+ N=12. The bars indicate mean value with standard deviation; Mixed-effect analysis; *p<0.05, **p<0.01, ***p<0.001, ****p<0.0001. **(E-H)** The morphology of rats’ kidneys on day 15 after cisplatin treatment of the following group: **(E)** healthy CTRL, **(F)** cisplatin CTRL, **(G)** cisplatin i.v. ABCB5+ and **(H)** cisplatin i.p. ABCB5+. H&E staining. Scale bars: 1 mm. Images acquired with Axio Scan.Z1 microscope (ZEISS). **(I–L)** Corresponding magnification of the corticomedullary region showing the altered cytoarchitecture due to the induced damage. Light blue arrows: proximal tubular dilatation; blue arrows: dregs of extruded necrotic cells; green arrows: protein casts; yellow arrows: new fibrotic tissue deposition and inflammatory cells infiltrate. H&E staining. Images acquired with Axio Scan.Z1 microscope (ZEISS).

Having shown that clinical-grade human ABCB5+ MSCs/their secreted naïve CM elicit both pro-regenerative and immunomodulatory effects in the xenotransplant context, we tested their therapeutic potential on a cisplatin-induced nephrotoxicity model in immunocompetent SD rats. The scheme of ABCB5+ MSC treatment is illustrated in **Figure 6A**. We chose to test a therapeutic setting where ABCB5+ MSCs were applied on day 3 post injury. Cisplatin toxicity was apparent as animals at day 2 post cisplatin treatment showed increased pCreatinine (plasma creatinine) and pUrea (plasma urea) levels as well as increased ABZWCY-HPβCD half-life values (**Figures 6B–D**) compared to the healthy control animals (39.93 ± 1.2 min), documenting cisplatin-mediated decreased GFR. Further, cisplatin-treated animals showed increased diuresis, loss of weight gain and reduced food intake starting from day 2, while water intake of cisplatin-treated animals was increased at day 2 and dropped from day 7 onwards (**Supplementary Table 3**). Cisplatin treatment resulted in acute kidney injury, which peaked on day 7 and lasted at least for 14 days after its administration.

Administration of human ABCB5+ MSCs at day 3 post-injury, either i.v. or i.p., caused no measurable attenuation of the cisplatin-induced kidney injury. There was no significant difference on pCreatinine, pUrea or other blood parameters observed between the cisplatin-ABCB5+ MSC group (both i.v. or i.p.) vs. the cisplatin vehicle group at any time points (day 7 and 14 after cisplatin administration) (**Figures 6B, C**; **Supplementary Table 3**).

Furthermore, cisplatin-treated animals showed marked histological changes compared to controls: dilated proximal tubuli in the cortical, inflammatory cell infiltrates in the juxtamedullary, and protein cast accumulations mostly in the papillary region (**Figures 6E–L**, **Supplementary Figure 3**). Animals that received ABCB5+ MSCs, either i.p. or i.v., did not show any visible histological differences from the cisplatin-treated animals. Image analysis indicated significantly more dilated tubuli in the cisplatin CTRL and cisplatin i.p. ABCB5+ group (**Supplementary Figure 3**). Hyaline cast formation was significantly higher in the cisplatin group. Inflammatory cell infiltrates were highly variable. Together, these data did not demonstrate a therapeutic benefit of either i.v.- or i.p.-administered ABCB5+ MSCs with regards to metabolic, urinary or plasmatic parameters and histology, when in injected on day 3 post injury.

### 3.5 ABCB5+ MSC grafting modulates mRNA expression patterns in cisplatin-induced nephrotoxicity

**Figure 7:**
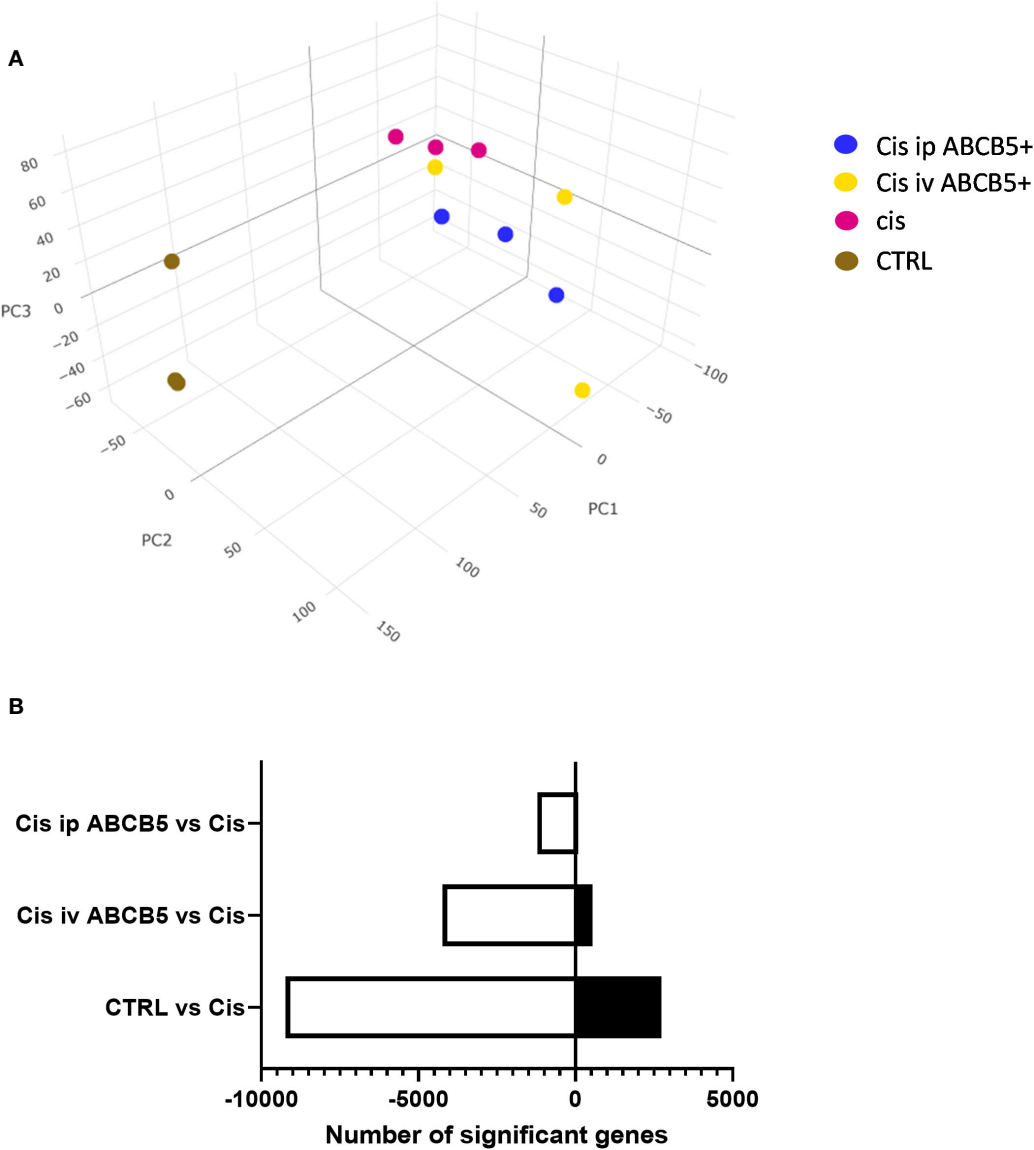
Cisplatin and ABCB5+ cells exert changes in gene expression of rats’ kidney. **(A)** The gene expression of rats’ kidney on day 15 after cisplatin treatment depicted in a 3D-principal component (PC) analysis graph. **(B)** The total number of genes that are significantly down- and upregulated in healthy CTRL, cisplatin i.v. and i.p. ABCB5+ groups, compared to cisplatin CTRL group. All groups N=3.

To investigate whether ABCB5+ MSC grafting modified renal gene expression, mRNA expression analysis of renal tissue of cisplatin- and ABCB5+ MSC-treated animals was performed with RNAseq technology. Cisplatin treatment clearly induced modification of gene expression profiles in the kidney as illustrated in 3D-principal component analysis (PCA) (**Figure 7A**). ABCB5+ MSC grafting, either via the i.v. or i.p. routes, modulated the cisplatin-induced gene expression changes. A difference between i.v. and i.p. administration of ABCB5+ MSCs also became apparent: the gene expression profiles of rats treated with ABCB5+ MSCs via the i.p. route clustered closer to each other than those subjected to i.v. ABCB5+ MSC administration. The number of genes that were affected significantly by the treatments also differed across the groups. When compared to the cisplatin-only group, healthy rats showed more than 8,000 and 2,500 upregulated and downregulated genes, respectively, implying that cisplatin drove expression changes in more than 10,000 genes (**Figure 7B**).

**Figure 8:**
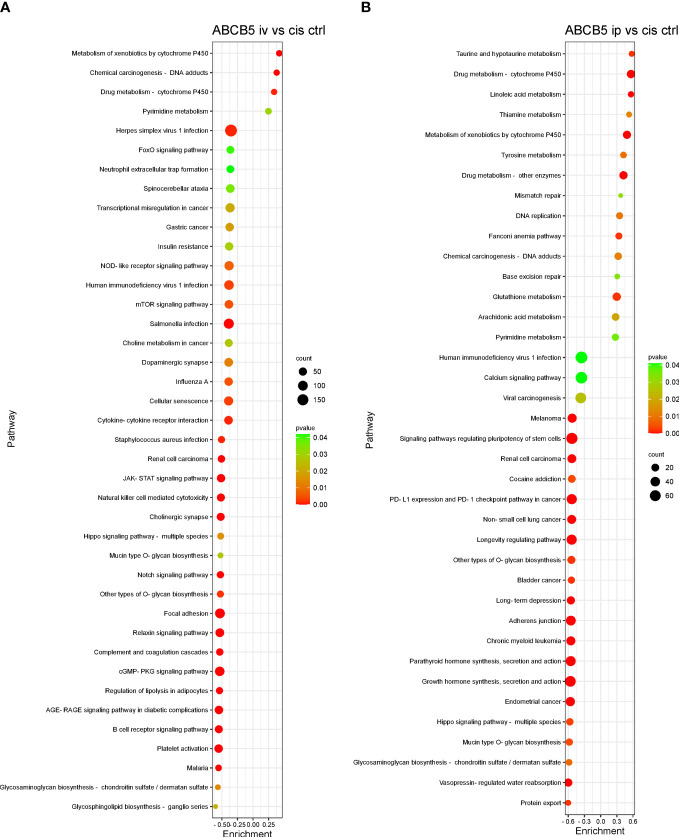
ABCB5+ cells promote changes in pathways of cisplatin-treated rats. Gene set enrichment analysis based on KEGG database shows the most upregulated and downregulated pathways of rats treated with cisplatin ABCB5+ cells iv **(A)** and ip **(B)**, in comparison with cisplatin CTRL. All groups N=3.

Some of the pathways with the highest normalized enrichment scores (NES), significantly affected by ABCB5+ MSC grafting in comparison with cisplatin-only are depicted in **Figures 8A, B**. Gene set enrichment analysis performed using the KEGG database showed 127 differentially expressed pathways in both the cisplatin + i.v. ABCB5+ MSC and the cisplatin + i.p. ABCB5+ MSC groups when compared to cisplatin only CTRL (**Supplementary Table 4**). Interestingly, despite differences in NES patterns between the i.v.- or i.p.-administered ABCB5+ MSC groups, the pathways involved in drug and xenobiotic metabolism were consistently and significantly upregulated in both groups (**Supplementary Table 4**). Other pathways that were consistently modulated by ABCB5+ MSC grafting regardless of the administration route were those involved in signal transduction, in the immune and endocrine system, in cancers, and in infectious diseases.

## 4 Discussion

In the last decades, MSCs have been used as a possible treatment for different types of kidney diseases, including cisplatin-induced AKI, where mostly encouraging results have been reported by using autologous, allogeneic, and xenogeneic cells (9). While our study addressing potential effects of clinical-grade human ABCB5+ MSCs in a therapeutic rat model of cisplatin-induced AKI revealed no clinical amelioration of kidney injury, this cell population exerted pro-regenerative and immunomodulatory effects on recipients at the mRNA expression level.

Outcomes of MSC therapy can be influenced by several variables, such as the experimental set up (MSC dose, time of the treatment, animal model) and the intrinsic factors of the transplanted cells. In cell therapy, MSC dose is crucial and can largely influence the outcome of an experiment. However, for defining a dose, many variables must be considered, such as cell type, administration route and time points, and animal model. Published MSC doses for the treatment of cisplatin-induced AKI in mice range between 5x10^5^ to 5x10^6^ cells/animal (9), i.e. approximately 25-250 x 10^6^ cells/kg assuming an average adult mouse weight of 20 g. Here, we chose a lower, more translationally relevant dose of 2x10^6^ cells/recipient rat (average weight in our study approximately 363 g, see **Supplementary Table 2**), i.e. grafts of approximately 5.51 x10^6^/kg. This 4.5- to 45-fold lower dosing strategy may have contributed to the observed differences in results, potentially warranting further pre-clinical rat dose-escalation studies to reach conclusions regarding comparative potential efficacy of this cell subset in this disorder.

The time point when cells are administered also plays an important role in the success rate of a potential treatment. In most studies in mice, MSCs were injected on the day or one day after cisplatin treatment either via the i.v. or via the i.p. route (44–46). We chose a therapeutic setting where ABCB5+ MSCs were injected on day 3 post injury. In this scenario, it is possible that injury will have been already too severe for the grafts to ameliorate clinical signs, whereas gene expression data document more subtle ABCB5+ MSC-mediated effects. Recent finding in a Macaca model support this notion. In this model the therapeutic effect of MSCs was only apparent when cells were administered in the early stage of the disease (47), suggesting that MSCs are active in preventing and attenuating cisplatin-induced injury, but ineffective once the injury has already progressed and established itself. Additionally, possibly further dosing might serve to overcome cisplatin toxicity, as suggested by Santeramo et al. (48). In this study, renal progenitor cell administration on day 2 and day 7 after cisplatin injection attenuated kidney injury. Furthermore, rats in this study were immunodeficient and not immunocompetent as in our study. As inflammation is one of the hallmarks of cisplatin-induced renal injury (6, 7), attempting to alleviate the injury in immune-competent animals as herein investigated might indeed be more challenging, yet also of greater translational relevance compared to studies in immune-deficient disease models (44, 48).

In this study, we did not track the cells after i.v. and i.p. injection. Accompanying data strongly support previous findings that MSCs are entrapped within the lungs, retained within the lung microvasculature, and rapidly cleared within the first one to three days (49, 50). So far, we were only able to track MSCs within a tissue when locally applied to the tissue, e,g, skin or muscle (51, 52). Given that we did not observe differences between i.v. and i.p. injection, we conclude that MSCs act via paracrine factors, strongly supported by our *in vitro* data, where already the conditioned medium from resting MSCs shows anti-apoptotic and anti-inflammatory activity.

Nevertheless, both i.v. and i.p. administered ABCB5+ MSCs induced upregulation of genes involved in xenobiotics degradation and metabolic pathways related to cytochrome p450 activity, which are downregulated by cisplatin treatment. Upregulation of cytochrome p450 expression by ABCB5+ MSCs might hereby reflect a response of proximal tubules to ABCB5+ MSCs to detoxify and excrete cisplatin (53). The degradation of xenobiotics involves biotransformation and is normally enzymatic, which mostly results in the formation of a hydrophilic compound different to the parent compound. This biotransformation can lead to either drug-drug interactions (54) or the excretion of compounds (53). Whether ABCB5+ MSC modify cisplatin metabolization was not elucidated in this study and warrants further investigation based on our results.

Some pathways in signal transduction have also been implicated in cisplatin-induced toxicity such as the nuclear factor kappa B (NF-κB), tumor necrosis factor (TNF), mitogen activated protein kinase (MAPK), PI3K-AKT (phosphatidylinositol 3-kinase (PI3K) and Akt/Protein Kinase B) and calcium signaling pathways. It has been shown that cisplatin can increase the NF-κB and TNF signaling pathways, which then contribute to further cell death and subsequent activation of the immune response in cisplatin-induced AKI (55, 56). In concordance to our data, cisplatin indeed upregulated these pathways, as inversely reflected by their downregulation in the healthy CTRL group compared to the cisplatin-only CTRL group. Importantly, ABCB5+ MSCs appeared to be capable of attenuating the effect of cisplatin on induction of NF-κB and TNF signaling. Similarly, the MAPK and calcium pathways were also attenuated by ABCB5+ MSC treatment. Interestingly, the upregulation of these signaling pathways is linked to more cell death and their inhibition has been reported to lead to amelioration of cisplatin toxicity (57, 58). Further, in line with and related to immune-relevant signal transduction pathways, a strong downregulation of the immune system was observed in the animals treated with ABCB5+ MSCs both via the i.v. and i.p. routes compared to cisplatin-only exposed controls. Given that cisplatin-induced nephrotoxicity also involves an exacerbated immune reaction (57), this downregulation may most likely be beneficial for kidney recovery. Taken together, the modulation of cisplatin-induced pathways by ABCB5+ MSC grafts implies that this cell population continues to hold promise for further investigations aimed at the prevention or improvement of cisplatin-induced AKI, following additional adjustment of dosing or timing strategies and additional relevant parameters.

Support to these pro-regenerative and immunomodulatory effects is provided by the herein reported *in vitro* data which document that ABCB5+ MSCs and their secretome reduce cisplatin-induced apoptosis of ciPTECs, inhibit mitogen-driven PBMC proliferation and modify macrophages responses, even in a xenogeneic context.

Because ABCB5+ MSCs showed similar therapeutic benefit to ASCs in cystic kidney disease in our previous study (16), we compared the effects of clinical-grade human ABCB5+ MSCs to human ASCs in our current models. Like ASCs, clinical-grade ABCB5+ MSCs, in this case even the secretome of unprimed/unlicensed MSCs, were capable of decreasing apoptosis of cisplatin-treated renal cells *in vitro*. Attenuation of apoptosis has been attributed to increased mitochondrial metabolism and anti-oxidative defenses of renal cells (45, 59). The observed upregulation in cell metabolism and signaling pathways in the kidneys of ABCB5+ MSC-grafted rats following cisplatin injury is consistent with these findings. Aiming to identify the mechanisms by which the MSC secretome protects from apoptosis, we applied a variety of inhibitors. Within a previous study, we observed that the content of free thiols within the ASC CM corresponded to its anti-apoptotic activity (Rendra et al. manuscript submitted). Comparing ABCB5+ and ASC CM, we found no free thiols in ABCB5+ CM, but variably high content in ASC CM. The entire absence of free thiol could thus not explain the 37% reduced apoptosis in ABCB5+ CM treated ciPTEC. NAC as ROS scavenger inhibited cisplatin-induced apoptosis indeed, but failed to boost the anti-oxidative/anti-apoptotic CM effect, as expected. We could not reproduce previous findings from Yuan et al. who documented that extracellular vesicles from induced pluripotent-derived MSCs protected from necroptosis caused by ischemia/reperfusion injury by delivering specificity protein (SP1) activating sphingosine kinase 1 expression (37). In our hands, neither extracellular vesicles were active (Rendra et al, manuscript submitted) nor did we observe profound necrosis/necroptosis in our system. Accordingly, we did not observe that SKI-II and Mit-A inhibited the anti-apoptotic activity of MSC CM. Both Nec-1 and SKI-II reduced the cisplatin-mediated apoptosis as shown before (60–62) by suppressing oxidative stress. In our study, SKI-II in addition significantly inhibited cisplatin-induced necrosis/necroptosis. We conclude that the mechanisms affected by the used inhibitors were at least not the main – and only- mechanism by which MSC CM acted in the system. The group of Darwin Prockop has shown that MSCs secrete stanniocalcin-1 that mediates anti-inflammatory, anti-apoptotic and anti-oxidative effects (38). However, bone marrow MSCs required licensing by cocultured UV-irradiated fibroblasts to secrete it. Given that we observed profound anti-apoptotic activity in CM derived from naïve MSCs, we postulated that ABCB5+ MSCs and ASCs could produce stanniocalcin even without challenge. Yet, a neutralizing antibody against stanniocalcin-1 mediated no effect at all in our conditions, supporting the notion that stanniocalcin-1 becomes secreted and active only upon priming/licensing. Continuing work of our group suggests that MSC CM modifies miRNA expression in cisplatin-injured ciPTEC and by this act anti-apoptotic (Scaccia et al, unpublished). Overall, our data suggest that MSC CM exerts its anti-apoptotic activity by different mechanisms, most likely culminating in suppressing oxidative stress and subsequent regulated cell death.

Since the immune system contributes to both cisplatin injury and interacts with MSCs even across species barriers in experimental disease models, we deemed it important to investigate how human ABCB5+ MSCs affect xenogeneic (rat) immune cells. In the pre-clinical setting, often conducted in xenogeneic transplantation models, it is critical to understand the peculiarities of cross-species immune modulation. Having observed that human ASCs fail to inhibit murine PBMC proliferation (17), we hypothesized that related phenomena may potentially affect therapeutic efficacy of human ABCB5+ MSCs in xenogeneic settings and lead to underestimation of their potential therapeutic capacity. First, our data reveal that ABCB5+ MSCs can suppress mitogen-driven proliferation of both human and rat PBMCs, while human ASCs were found to exert this capacity only in the human context. The inhibitory capacity of human ABCB5+ MSCs acting on human PBMCs was comparably lower than that of ASCs in the herein described assay systems. The fact that clinical-grade ABCB5+ MSCs were freshly thawed from cryopreserved batches whereas ASCs were directly derived from cell cultures is not deemed responsible for the observed differential effects, as cryopreserved ABCB5+ MSCs have been validated to fully retain the phenotype and function of freshly isolated ABCB5+ MSCs. While the anti-proliferative effects on human PBMCs exerted by human ASCs are highly dependent on the mediator IDO (9, 17) (reproduced in this study), ABCB5+ MSCs utilize rather different effector molecules to inhibit PBMC proliferation, which include the negative co-stimulatory pathway members PD-1 and CD86 (B7-2) (13).

Second, our data suggest that ABCB5+ MSCs, in particular ABCB5+MSC-derived naïve CM, affected macrophage polarization to a lower degree than ASC CM. Apparently, ABCB5+ MSCs require cell interaction to prime their anti-inflammatory secretome (15). In fact, IL-1RA secretion after coculture with M1-polarized human macrophages (63, 64) is tested and validated for each ABCB5+ cell batch release as a predictive measure of the anti-inflammatory potency in M1 macrophage-dominated inflammatory milieu. The evaluation of the cells’ actual biological functionality using appropriate tests that predict their clinical effectiveness is a vital component of the quality assessment of cell therapy products.

Another possibility potentially contributing to our results, which cannot be ruled out, is the potential accelerated rejection of administrated MSCs. Although certain MSCs, like ABCB5+ MSCs, are relatively immune-privileged (13), some recent studies have shown that other MSC types can be recognized and rejected by the recipient’s immune response (65, 66). Additionally, transplantation of other MSC types, especially in human leukocyte antigen (HLA) mis-matched allogeneic settings, can sometimes elicit the production of antibodies against MSCs (67–69).

## 5 Conclusion

In this study, clinical-grade human ABCB5+ MSCs exerted significant inhibitory effects on PBMC proliferation, macrophage TNF-α secretion, and apoptotic cell death of renal cells *in vitro*, even in a xenogeneic context. Additionally, despite an apparent lack of amelioration of renal damage at physiologic, metabolic and histologic levels *in vivo*, ABCB5+ MSC xenotransplantation exerted significant modulatory effects on recipient mRNA expression patterns toward an anti-inflammatory and pro-regenerative state. These results demonstrate anti-inflammatory and pro-regenerative functions of clinical grade ABCB5+ MSCs and provide a rationale to assess, following administration dose and timing modifications, the therapeutic utility of this cell population further for the treatment and/or prevention of cisplatin chemotherapy-induced organ toxicity.

## Data availability statement

The datasets presented in this study can be found in online repositories. The names of the repository/repositories and accession number(s) can be found below: Expression Omnibus database via accession numbers GSE140601 and GSE223675.

## Ethics statement

The studies involving humans were approved by Mannheim Ethics Commission II, Mannheim, Germany. The studies were conducted in accordance with the local legislation and institutional requirements. The participants provided their written informed consent to participate in this study. The animal study was approved by Regierungspräsidium Karlsruhe, Germany. The study was conducted in accordance with the local legislation and institutional requirements.

## Author contributions

ER, AT, CD, NG, and KB contributed to the conception and design of the study. ER and AT performed research, data analysis and interpretation of *in vitro* experiments. CD conducted research, data analysis and interpretation of *in vivo* experiments. CS performed bioinformatic analysis and interpretation. NG and KB contributed to data interpretation as well as provided administrative and financial support. MK and CG provided financial support by cell provision and administrative support. MF conducted ABCB5 monoclonal antibody generation, ABCB5+ cell isolation strategy design and data analysis and interpretation. All authors contributed to the article and approved the submitted version.

## Glossary

**Table:**

ABCB5	ATP-binding cassette member B5
ABCB5+	ABCB5 positive
AKI	acute kidney disease
ALT	alanine aminotransferase
Anti-STC-1	anti-stanniocalcin-1
ASC	adipose stromal cell
ASCs	adipose stromal cells
AST	aspartate aminotransferase
BW	body weight
ciPTEC	conditionally immortalized proximal tubular epitelial cell
ciPTECs	conditionally immortalized proximal tubular epitelial cells
Cis	cisplatin
CKD	chronic kidney disease
CM	conditioned medium
ConA	Concanavalin A
CTRL	control
DIRCs	dermal immune-regulatory cells
DMEM	Dulbecco´s Modified Eagle Medium
DMSO	Dimethyl sulfoxide
DPBS	Dulbecco’s phosphate-buffered saline
ECM	extracellular matrix
EDTA	Ethylenediaminetetraacetic acid
ESRD	end-stage renal disease
EVs	Extracellular vesicles
FACS	flow assisted cell sorting
GAG	glycosaminoglycan
GCU	green calibrated units
GFR	glomerual filtration rate
GGT	γ-glutamyltransferase
GLDH	glutamate dehydrogenase
GSEA	Gene set enrichment analysis
H&E	hematoxylin and eosin
HBSS	Hanks’
HLA	human leukocyte antigen
hMac	human macrophages
hMSCs	human mesenchymal stromal cells
hPBMCs	human peripheral blood mononuclear cells
hTNF-α	human tumor necrosis factor-α
IDO	indoleamine 2,3-dioxygenase
IL-1RA	interleukin-1 receptor antagonist
IL-2	Interleukin-2
IL-6	Interleukin-6
i.p.	intraperitoneal
i.v.	intravenous
KEGG	Kyoto Encyclopedia of Genes and Genomes
Kyn	kynurenine
LPS	lipopolysaccharide
MACS	Microbeads and magnetic activated cell sorting
MCP-1	monocyte chemoattractant protein-1
MCSF	macrophage colony-stimulating factor
MHC	major histocompatibility complex
Mit-A	mithramycin A
mRNA	messenger RNA
MSC	mesenchymal stromal cell
MSCs	mesenchymal stromal cells
n	number of technical replicates
N	number of biological replicates
NAC	N-acetylcysteine
Nec-1	necrostain-1
NFκB	nuclear factor kappa B
NO	nitric oxide
NOS	nitric oxide synthase
OD	optical density
PBMC	peripheral blood mononuclear cells
PD-1	programmed cell death 1
PFA	paraformaldehyde
PGE-2	prostaglandin E-2
PHA	phytohemagglutinin
rMac	rat macrophages
rMSC	rat mesenchymal stromal cell
rMSCs	rat mesenchymal stromal cells
RNAseq	RNA sequencing
rPBMCs	rat peripheral blood mononuclear cells
RPMI	Roswell Park Memorial Institute
rTNF-α	rat tumor necrosis factor-α
SD	Sprague Dawley
SFM	serum free medium
SKI-II	sphingosine kinase inhibitor
SN	supernatant
TCA	trichloroacetic acid
TGF-β1	transforming growth factor- β1
TNF-α	tumor necrosis factor-α
TSG-6	tumor necrosis factor-inducible gene 6 protein
