# Supplementary material for: Clinical-grade human skin-derived ABCB5+ mesenchymal stromal cells exert anti-apoptotic and anti-inflammatory effects in vitro and modulate mRNA expression in a cisplatin-induced kidney injury murine model
Source: Front Immunol. 2024 Jan 11;14:1228928. doi: 10.3389/fimmu.2023.1228928 (PMC10808769; doi:10.3389/fimmu.2023.1228928)
Supplement: Supplementary file 1 [file DataSheet_1.pdf]

Supplementary Material

Clinical-grade human skin-derived ABCB5+ mesenchymal stromal cells exert modulatory functions on mRNA expression in a cisplatin-induced kidney injury murine model

Erika Rendra [1], Adriana Torres Crigna [1], Cristina Daniele [2], Carsten Sticht [2], Maike Cueppers [1], Mark Andreas Kluth [3], Christoph Ganss, Markus H. Frank [4,5,6,7], Norbert Gretz [2], Karen Bieback *[1,8]

*** Correspondence:** Karen Bieback, Dr. rer.nat.: Karen.Bieback@medma.uni-heidelberg.de

Supplementary Table 1. List of antibodies used for immunophenotyping of human MSCs

Antibody	Clone	Company
**CD202b-AF488**	33.1	BioLegend, San Diego, USA
**CD73-PE**	AD2	BioLegend, San Diego, USA
**CD90-APC**	5E10	BD Biosciences, Heidelberg, Germany
**CD44-APC Cy7**	IM7	BioLegend, San Diego, USA
**CD31-FITC**	WM59	BD Biosciences, Heidelberg, Germany
**CD34-APC**	581	BD Biosciences, Heidelberg, Germany
**CD146-PE**	TEA1/34	Beckman Coulter, California, USA
**CD45-PE-Cy7**	HI30	BioLegend, San Diego, USA
**NG-2-AlexaFluor 488**	9.2.27	eBioscience, California, USA
**CD140a-PE**	16A1	BioLegend, San Diego, USA
**CD140b-APC**	18A2	BioLegend, San Diego, USA
**CD105-PE Cy7**	SN6	eBioscience, California, USA
**CD13-APC Cy7**	WM15	BioLegend, San Diego, USA
**HLA-ABC-PE Vio770**	REA230	Miltenyi Biotec, Bergisch Gladbach, Germany
**HLA-DR-APC Cy7**	L243	BioLegend, San Diego, USA
**CD106-FITC**	51-10C9	BD Biosciences, Heidelberg, Germany
**CD248-AF647**	B1/35	BD Biosciences, Heidelberg, Germany
**CD49a-AF647**	TS2/7	AbD Serotec, Feldkirchen, Germany
**CD49f-PE**	GoH3	BioLegend, San Diego, USA
**CD29-AlexaFluor 488**	TS2/16	BioLegend, San Diego, USA
**CD49b-FITC**	P1E6-C5	BioLegend, San Diego, USA
**CD49d-PE Cy7**	9F10	BioLegend, San Diego, USA
**CD49e-PE**	NKI-SAM-1	BioLegend, San Diego, USA
**Integrinß7-APC**	FIB504	BioLegend, San Diego, USA

Supplementary Table 2. Changes in body weight, diuresis, food and water intake before and after metabolic cage allocation.
Control N=3; cisplatin N=15; iv ABCB5+ N=12; ip ABCB5+ N=11; vehicle N=3. Data are shown as means ± SD. Values significantly different from cisplatin CTRL are indicated as *p< 0.05 and **p< 0.005.

Parameter	Group	Baseline	Day 2	Day 7	Day 14
**Diuresis (ml/16h)**	Healthy CTRL	10.93 ± 1.9	10.5 ± 1.1	11.86 ± 1.1	14.50 ± 2.3
	Cisplatin CTRL	11.68 ± 2.0	16.18 ± 10.8	36.03 ± 10.0	37.19 ± 9.9
	Cisplatin Vehicle CTRL	14.13 ± 3.6	12.63 ± 3.9	39.80 ± 6.6	36.67 ± 7.4
	Cisplatin-iv ABCB5+	13.55 ± 3.5	16.38 ± 13.5	40.50 ± 15.3	43.10 ± 7.3
	Cisplatin-ip ABCB5+	10.85 ± 1.7	16.81 ± 12.8	35.85 ± 3.8	38.18 ± 4.6
**Water intake (ml/16h)**	Healthy CTRL	30.83 ± 1.8	34.26 ± 2.0	33.90 ± 2.9	29.30 ± 5.9
	Cisplatin CTRL	26.38 ± 2.9	23.38 ± 16.8	56.35 ± 18.7	55.75 ± 12.5
	Cisplatin Vehicle CTRL	25.00 ± 7.9	18.80 ± 4.13	64.47 ± 5.7	60.90 ± 11.9
	Cisplatin-iv ABCB5+	29.16 ± 4.7	21.50 ± 15.3	60.27 ± 13.4	61.09 ± 9.3
	Cisplatin-ip ABCB5+	25.50 ± 4.2	22.44 ± 13.6	51.69 ± 6.3	60.06 ± 6.8
**Body weight (g)**	Healthy CTRL	360.33 ± 41.3	409.83 ± 22.0	435.33 ± 12.5**	455.33 ± 18.0**
	Cisplatin CTRL	366.53 ± 33.5	395.47 ± 37.1	376.80 ± 49.9	386.27 ± 53.3
	Cisplatin Vehicle CTRL	377.33 ± 33.0	378.67 ± 48.7	360.33 ± 49.9	384.33 ± 58.1
	Cisplatin-iv ABCB5+	371.41 ± 32.7	375.25 ± 53.7	340.25 ± 59.4	361.50 ± 63.7
	Cisplatin-ip ABCB5+	339.00 ± 39.7	358.91 ± 47.2	328.73 ± 54.9	355.45 ± 57.9
**Food intake (g/16h)**	Healthy CTRL	20.96 ± 5.2	26.26 ± 2.6	24.86 ± 1.4	22.43 ± 4.6
	Cisplatin CTRL	19.54 ± 3.1	8.93 ± 5.8	15.87 ± 5.3	21.39 ± 3.0
	Cisplatin Vehicle CTRL	22.80 ± 3.4	9.70 ± 0.6	17.17 ± 3.0	22.00 ± 2.7
	Cisplatin-iv ABCB5+	21.69 ± 4.6	9.55 ± 3.5	15.47 ± 4.9	21.69 ± 3.2
	Cisplatin-ip ABCB5+	20.04 ± 3.7	8.77 ± 1.5	15.68 ± 3.9	22.14 ± 3.0

Supplementary Table 3. Changes in plasma biochemistry

Control N=3; cisplatin N=15; iv ABCB5+ N=12; ip ABCB5+ N=11; Vehicle N=3. Data are shown as means ± SD. Values significantly different from cisplatin CTRL are indicated as *p< 0.05 and **p< 0.005

Parameter	Group	Baseline	Day 2	Day 7	Day 14
	Healthy CTRL	153.66 ± 6.1	161.33 ± 4.6	154.33 ± 8.9	153.33 ± 8.3
	Cisplatin CTRL	149.06 ± 7.8	158.66 ± 18.2	160.33 ± 13.1	142.53 ± 6.9
**Glucose (mg/dl)**	Cisplatin Vehicle CTRL	159.33 ± 9.9	159.33 ± 15.6	147.67 ± 8.9	155.33 ± 5.7
	Cisplatin-iv ABCB5+	158.08 ± 18.1	164.08 ± 15.0	150.17 ± 18.5	154.67 ± 12.1*
	Cisplatin-ip ABCB5+	173.18 ± 38.0	153.72 ± 6.9	156.00 ± 18.5	141.09 ± 6.47
	Healthy CTRL	55.66 ± 1.5	59.33 ± 2.3	59.00 ± 2.0	58.33 ± 1.5
	Cisplatin CTRL	55.53 ± 1.8	60.26 ± 2.1	55.66 ± 2.9	56.26 ± 3.1
**Protein (mg/dl)**	Cisplatin Vehicle CTRL	58.33 ± 2.1	59.00 ± 4.3	54.33 ± 2.1	55.33 ± 4.7
	Cisplatin-iv ABCB5+	55.50 ± 2.5	59.41 ± 1.9	55.75 ± 2.9	57.92 ± 2.3
	Cisplatin-ip ABCB5+	56.72 ± 2.4	60.81 ± 2.7	54.54 ± 2.4	56.91 ± 1.7
	Healthy CTRL	97.66 ± 28.4	100.00 ± 28.2	88.33 ±18.4	87.66 ± 13.0
	Cisplatin CTRL	96.00 ± 12.5	140.20 ± 21.5	113.20 ± 24.3	117.93 ± 15.7
**Cholesterol (mg/dl)**	Cisplatin Vehicle CTRL	88.00 ± 8.5	135.67 ± 18.44	117.67 ± 21.4	122.00 ± 23.4
	Cisplatin-iv ABCB5+	100.17 ± 10.5	142.42 ± 11.1	118.17 ± 13.9	129.58 ± 22.5
	Cisplatin-ip ABCB5+	94.18 ± 14.0	148.36 ± 21.3*	105.82 ± 17.3	114.00 ± 17.2
	Healthy CTRL	90.00 ± 34.6	136.00 ± 26.2	121.66 ± 39.8	108.66 ± 30.4
	Cisplatin CTRL	94.40 ± 26.5	73.33 ± 27.0	130.00 ± 37.8	127.33 ± 38.8
**Triglycerides (mg/dl)**	Cisplatin Vehicle CTRL	151.67 ± 57.9	61.67 ± 12.7	156.67 ± 55.7	84.33 ± 21.5
	Cisplatin-iv ABCB5+	115.58 ± 37.3	66.92 ± 11.1	124.67 ± 33.5	127.83 ± 33.1
	Cisplatin-ip ABCB5+	118.00 ± 39.7	68.18 ± 10.0	127.18 ± 50.3	148.73 ± 45.3
	Healthy CTRL	48.3 ± 11.2	67.00 ± 17.5	54.00 ± 19.5	55.00 ± 5.2
**AST (U/l)**	Cisplatin CTRL	52.8 ± 11.5	39.92 ± 6.7	61.93 ± 35.8	51.20 ± 20.8
	Cisplatin Vehicle CTRL	78.00 ± 6.9	52.33 ± 10.2	76.33 ± 15.9	68.33 ± 33.2
	Cisplatin-iv ABCB5+	54.00 ± 12.7	37.83 ± 7.0	47.92 ± 16.2	49.17 ± 27.4

	Cisplatin-ip ABCB5+	64.45 ± 15.0	43.36 ± 7.7	74.45 ± 25.7	58.27 ± 18.0
**ALT (U/l)**	Healthy CTRL	103.00 ± 11.9	92.33 ± 2.8	92.33 ± 14.0	100.00 ± 26.8
	Cisplatin CTRL	100.53 ± 5.3	104.46 ± 12.7	97.60 ±18.5	91.13 ± 14.2
	Cisplatin Vehicle CTRL	88.67 ± 9.9	150.33 ± 50.6	125.67 ± 37.2	101.33 ± 17.7
	Cisplatin-iv ABCB5+	88.08 ± 14.9	96.75 ± 16.1	90.17 ± 20.0	98.75 ± 30.6
	Cisplatin-ip ABCB5+	97.73 ± 27.1	108.81 ± 14.9	98.64 ± 13.9	92.70 ± 13.5
**GGT (U/l)**	Healthy CTRL	3.00 ± 0.0	3.00 ± 0.0	3.00 ± 0.4	3.00 ± 0.0
	Cisplatin CTRL	3.00 ± 0.0	3.66 ± 0.9	3.26 ± 0.1	3.00 ± 0.0
	Cisplatin Vehicle CTRL	3.00 ± 0.0	3.00 ± 0.0	2.67 ± 0.6	3.00 ± 0.0
	Cisplatin-iv ABCB5+	3.00 ± 0.0	3.00 ± 0.0	3.17 ± 1.3	3.00 ± 0.0
	Cisplatin-ip ABCB5+	3.00 ± 0.0	3.00 ± 0.0	3.54 ± 1.3	3.63 ± 1.3*
**GLDH (U/l)**	Healthy CTRL	8.22 ± 0.8	7.80 ± 0.1	7.68 ± 1.2	8.31 ± 2.2
	Cisplatin CTRL	8.60 ± 1.3	9.13 ± 2.1	19.2 ± 4.5	4.91 ± 1.2
	Cisplatin Vehicle CTRL	6.22 ± 2.0	8.33 ± 1.6	11.99 ± 4.5	6.45 ± 0.7
	Cisplatin-iv ABCB5+	7.50 ± 3.5	9.22± 1.9	19.94 ± 30.2	6.48 ± 2.7
	Cisplatin-ip ABCB5+	6.60 ± 1.4	9.74 ± 1.6	16.72 ± 16.5	6.51 ± 3.9
**Na (mmol/l)**	Healthy CTRL	133.66 ± 6.4	141.00 ± 1.0	136.66 ± 7.5	143.50 ± 2.1
	Cisplatin CTRL	139.53 ± 4.5	140.13 ± 1.5	138.00 ± 4.2	139.36 ± 2.7
	Cisplatin Vehicle CTRL	143.67 ± 2.9	140.33 ± 2.1	139.67 ± 1.5	140.00 ± 2.6
	Cisplatin-iv ABCB5+	142.75 ± 1.4	140.75 ± 1.2	139.33 ± 2.2	142.00 ± 3.1
	Cisplatin-ip ABCB5+	142.45 ± 0.9	141.82 ± 1.2**	141.64 ± 4.4*	141.45 ± 2.5
**Ca (mmol/l)**	Healthy CTRL	2.71 ± 0.0	2.80 ± 0.1	2.72 ± 0.0	2.71 ± 0.0
	Cisplatin CTRL	2.71 ± 0.0	2.73 ± 0.0	4.75 ± 7.6	2.84 ± 0.0
	Cisplatin Vehicle CTRL	2.76 ± 0.0	2.73 ± 0.0	2.84 ± 0.1	2.69 ± 0.1
	Cisplatin-iv ABCB5+	2.71 ± 0.1	2.72 ± 0.0	2.89 ± 0.2	2.88 ± 0.1
	Cisplatin-ip ABCB5+	2.71 ± 0.1	2.75 ± 0.1	2.80 ± 0.1	2.80 ± 0.1
**K (mmol/l)**	Healthy CTRL	4.72 ± 0.5	4.97 ± 0.3	4.41 ± 0.4	4.45 ± 0.6
	Cisplatin CTRL	4.95 ± 0.3	4.19 ± 0.3	4.77 ± 0.5	5.55 ± 0.6

	Cisplatin Vehicle CTRL	5.14 ± 0.3	4.26 ± 0.2	4.94 ± 0.2	5.77 ± 0.7
	Cisplatin-iv ABCB5+	5.20 ± 0.3	4.14 ± 0.2	5.34 ± 0.4*	5.90 ± 0.6
	Cisplatin-ip ABCB5+	5.37 ± 0.4	4.28 ± 0.2	5.08 ± 0.4	5.43 ± 0.5
**PO4 (mmol/l)**	Healthy CTRL	2.55 ± 0.3	2.49 ± 0.1	2.31 ± 0.2	2.48 ± 0.1
	Cisplatin CTRL	2.75 ± 0.1	2.47 ± 0.2	2.20 ± 0.9	1.41 ± 0.5
	Cisplatin Vehicle CTRL	2.33 ± 0.3	2.40 ± 0.3	1.74 ± 0.2	1.61 ± 0.6
	Cisplatin-iv ABCB5+	2.51 ± 0.3	2.46 ± 0.2	2.07 ± 1.0	1.32 ± 0.3
	Cisplatin-ip ABCB5+	2.57 ± 0.4	2.51 ± 0.2	1.89 ± 0.6	1.34 ± 0.6

Supplementary Table 4. ABCB5+ cells exert modulatory function in cisplatin-treated rats' kidneys

GSEA analysis RNA sequencing using KEGG database sorted by subcategory. Significant data (p-value< 0.05) differentially expressed pathways (healthy control or treatment vs cisplatin control); for each pathway the normalized enrichment score (NES) is given. Downregulated pathways are displayed in green (light green: -1.5 >NES> 0; green: -2 >NES>-1.5; dark green: NES >-2), upregulated pathways in red (light red: 1.5 >NES> 0; red: 2 >NES> 1.5; dark red: NES >2). All groups N=3.

Description	Sub.Category	Healthy vs Cisplatin Control		ABCB5 iv vs Cisplatin Control		ABCB5 ip vs Cisplatin Control	
		NES	p-Value	NES	p-Value	NES	p-Value
Pyrimidine metabolism	1.4. Nucleotide metabolism	1.31	0.097	1.46	0.031	1.34	0.036
Lysine degradation	1.5. Amino acid metabolism	2.48	0.000	-1.43	0.018	-1.53	0.004
Glycosaminoglycan biosynthesis - chondroitin sulfate / dermatan sulfate	1.7. Glycan biosynthesis and metabolism	-1.57	0.034	-1.59	0.014	-1.6	0.008
Mucin type O-glycan biosynthesis	1.7. Glycan biosynthesis and metabolism	-0.8	0.758	-1.53	0.027	-1.64	0.004
Other types of O-glycan biosynthesis	1.7. Glycan biosynthesis and metabolism	-1.04	0.428	-1.65	0.002	-1.65	0.002
Drug metabolism - cytochrome P450	1.11. Xenobiotics biodegradation and metabolism	3.22	0.000	1.83	0.001	2.68	0.000
Metabolism of xenobiotics by cytochrome P450	1.11. Xenobiotics biodegradation and metabolism	3.02	0.000	2.38	0.000	2.31	0.000
Protein processing in endoplasmic reticulum	2.3. Folding, sorting and degradation	-0.64	0.997	-1.6	0.000	-1.8	0.000
Calcium signaling pathway	3.2. Signal transduction	-0.99	0.502	-1.43	0.000	-1.23	0.041
FoxO signaling pathway	3.2. Signal transduction	-1.27	0.094	-1.27	0.041	-1.41	0.006
NF-kappa B signaling pathway	3.2. Signal transduction	-2.15	0.000	-1.6	0.000	-1.42	0.007
Phospholipase D signaling pathway	3.2. Signal transduction	-1.52	0.004	-1.63	0.000	-1.42	0.006
cAMP signaling pathway	3.2. Signal transduction	-1.22	0.117	-1.58	0.000	-1.43	0.001
HIF-1 signaling pathway	3.2. Signal transduction	-1.57	0.005	-1.49	0.001	-1.45	0.003
Apelin signaling pathway	3.2. Signal transduction	-1.48	0.007	-1.59	0.000	-1.45	0.003
Ras signaling pathway	3.2. Signal transduction	-1.69	0.000	-1.48	0.000	-1.49	0.000
VEGF signaling pathway	3.2. Signal transduction	-1.62	0.003	-1.5	0.009	-1.53	0.005
Sphingolipid signaling pathway	3.2. Signal transduction	-1.61	0.002	-1.51	0.000	-1.53	0.000

JAK-STAT signaling pathway	3.2. Signal transduction	-1.75	0.000	-1.79	0.000	-1.55	0.000
TGF-beta signaling pathway	3.2. Signal transduction	-1.28	0.094	-1.49	0.003	-1.57	0.000
Hedgehog signaling pathway	3.2. Signal transduction	-1.28	0.126	-1.42	0.022	-1.58	0.002
PI3K-Akt signaling pathway	3.2. Signal transduction	-1.78	0.000	-1.62	0.000	-1.59	0.000
mTOR signaling pathway	3.2. Signal transduction	-0.99	0.514	-1.36	0.005	-1.6	0.000
Notch signaling pathway	3.2. Signal transduction	-1.66	0.002	-1.72	0.000	-1.62	0.001
TNF signaling pathway	3.2. Signal transduction	-2.09	0.000	-1.61	0.000	-1.64	0.000
MAPK signaling pathway	3.2. Signal transduction	-1.88	0.000	-1.55	0.000	-1.66	0.000
Hippo signaling pathway - multiple species	3.2. Signal transduction	-1.75	0.002	-1.56	0.015	-1.66	0.003
Wnt signaling pathway	3.2. Signal transduction	-1.36	0.027	-1.39	0.003	-1.68	0.000
Rap1 signaling pathway	3.2. Signal transduction	-1.77	0.000	-1.68	0.000	-1.68	0.000
cGMP-PKG signaling pathway	3.2. Signal transduction	-1.26	0.098	-1.88	0.000	-1.68	0.000
ErbB signaling pathway	3.2. Signal transduction	-1.51	0.015	-1.34	0.035	-1.72	0.000
Hippo signaling pathway	3.2. Signal transduction	-1.68	0.000	-1.42	0.001	-1.76	0.000
ECM-receptor interaction	3.3. Signaling molecules and interaction	-1.78	0.000	-1.66	0.000	-1.32	0.046
Endocytosis	4.1. Transport and catabolism	-1.48	0.001	-1.47	0.000	-1.41	0.000
Cellular senescence	4.2. Cell growth and death	-1.77	0.000	-1.39	0.003	-1.31	0.012
Gap junction	4.3. Cellular community - eukaryotes	-1.71	0.001	-1.67	0.000	-1.7	0.000
Focal adhesion	4.3. Cellular community - eukaryotes	-1.87	0.000	-1.89	0.000	-1.72	0.000
Signaling pathways regulating pluripotency of stem cells	4.3. Cellular community - eukaryotes	-1.4	0.028	-1.45	0.002	-1.79	0.000
Adherens junction	4.3. Cellular community - eukaryotes	-1.61	0.003	-1.54	0.002	-1.79	0.000
Regulation of actin cytoskeleton	4.5. Cell motility	-1.88	0.000	-1.65	0.000	-1.79	0.000
Fc gamma R-mediated phagocytosis	5.1. Immune system	-2.01	0.000	-1.54	0.001	-1.38	0.014
Fc epsilon RI signaling pathway	5.1. Immune system	-1.91	0.000	-1.62	0.001	-1.4	0.029
Th17 cell differentiation	5.1. Immune system	-1.99	0.000	-1.48	0.002	-1.42	0.005
Natural killer cell mediated cytotoxicity	5.1. Immune system	-2.11	0.000	-1.74	0.000	-1.45	0.007
Chemokine signaling pathway	5.1. Immune system	-2.01	0.000	-1.72	0.000	-1.47	0.000

Th1 and Th2 cell differentiation	5.1. Immune system	-1.99	0.000	-1.45	0.005	-1.49	0.004
T cell receptor signaling pathway	5.1. Immune system	-1.94	0.000	-1.62	0.000	-1.51	0.002
C-type lectin receptor signaling pathway	5.1. Immune system	-1.98	0.000	-1.69	0.000	-1.52	0.001
Leukocyte transendothelial migration	5.1. Immune system	-1.87	0.000	-1.57	0.000	-1.54	0.000
Platelet activation	5.1. Immune system	-1.9	0.000	-1.92	0.000	-1.54	0.000
Toll-like receptor signaling pathway	5.1. Immune system	-2.07	0.000	-1.47	0.004	-1.56	0.001
B cell receptor signaling pathway	5.1. Immune system	-2.17	0.000	-1.85	0.000	-1.59	0.001
Insulin secretion	5.2. Endocrine system	-1.23	0.156	-1.46	0.007	-1.43	0.010
Renin secretion	5.2. Endocrine system	-0.96	0.552	-1.56	0.002	-1.45	0.019
Oxytocin signaling pathway	5.2. Endocrine system	-1.54	0.002	-1.61	0.000	-1.47	0.001
Relaxin signaling pathway	5.2. Endocrine system	-1.75	0.000	-1.85	0.000	-1.51	0.001
Regulation of lipolysis in adipocytes	5.2. Endocrine system	-0.92	0.587	-1.74	0.000	-1.52	0.008
Thyroid hormone synthesis	5.2. Endocrine system	-1.02	0.454	-1.57	0.002	-1.55	0.003
Cortisol synthesis and secretion	5.2. Endocrine system	-1.13	0.293	-1.57	0.003	-1.56	0.003
GnRH signaling pathway	5.2. Endocrine system	-1.55	0.009	-1.41	0.015	-1.64	0.000
Insulin signaling pathway	5.2. Endocrine system	-1.31	0.072	-1.41	0.002	-1.65	0.000
Thyroid hormone signaling pathway	5.2. Endocrine system	-1.16	0.213	-1.45	0.002	-1.65	0.000
Prolactin signaling pathway	5.2. Endocrine system	-1.6	0.005	-1.42	0.020	-1.68	0.000
Estrogen signaling pathway	5.2. Endocrine system	-1.45	0.033	-1.6	0.000	-1.69	0.000
Melanogenesis	5.2. Endocrine system	-1.35	0.054	-1.6	0.000	-1.7	0.000
Parathyroid hormone synthesis, secretion and action	5.2. Endocrine system	-1.18	0.200	-1.65	0.000	-1.85	0.000
Vascular smooth muscle contraction	5.3. Circulatory system	-1.18	0.183	-1.66	0.000	-1.44	0.004
Adrenergic signaling in cardiomyocytes	5.3. Circulatory system	-1.37	0.031	-1.46	0.001	-1.46	0.001
Dopaminergic synapse	5.6. Nervous system	-1.24	0.128	-1.36	0.012	-1.4	0.007
Neurotrophin signaling pathway	5.6. Nervous system	-1.94	0.000	-1.42	0.003	-1.55	0.000
Cholinergic synapse	5.6. Nervous system	-1.71	0.001	-1.77	0.000	-1.56	0.000
Long-term potentiation	5.6. Nervous system	-1.18	0.231	-1.52	0.005	-1.57	0.001
Long-term depression	5.6. Nervous system	-1.12	0.301	-1.48	0.012	-1.74	0.000
Osteoclast differentiation	5.8. Development	-2.24	0.000	-1.72	0.000	-1.43	0.004
Axon guidance	5.8. Development	-1.79	0.000	-1.53	0.000	-1.74	0.000
Longevity regulating pathway - multiple species	5.9. Aging	1	0.440	-1.48	0.007	-1.58	0.003

Longevity regulating pathway	5.9. Aging	-1.3	0.085	-1.52	0.002	-1.76	0.000
Chemical carcinogenesis - DNA adducts	6.1. Cancers: Overview	2.96	0.000	2.1	0.000	1.55	0.012
Viral carcinogenesis	6.1. Cancers: Overview	-1.89	0.000	-1.42	0.000	-1.26	0.025
Transcriptional misregulation in cancer	6.1. Cancers: Overview	-1.73	0.000	-1.3	0.021	-1.34	0.010
Choline metabolism in cancer	6.1. Cancers: Overview	-0.95	0.568	-1.34	0.027	-1.45	0.006
Pathways in cancer	6.1. Cancers: Overview	-1.45	0.000	-1.47	0.000	-1.47	0.000
Proteoglycans in cancer	6.1. Cancers: Overview	-1.89	0.000	-1.62	0.000	-1.67	0.000
MicroRNAs in cancer	6.1. Cancers: Overview	-1.93	0.000	-1.48	0.000	-1.76	0.000
Small cell lung cancer	6.2. Cancers: Specific types	-1.62	0.002	-1.36	0.021	-1.42	0.011
Colorectal cancer	6.2. Cancers: Specific types	-1.61	0.002	-1.51	0.002	-1.45	0.005
Glioma	6.2. Cancers: Specific types	-1.74	0.000	-1.45	0.009	-1.47	0.005
Gastric cancer	6.2. Cancers: Specific types	-1.49	0.007	-1.31	0.018	-1.57	0.000
Acute myeloid leukemia	6.2. Cancers: Specific types	-1.9	0.000	-1.57	0.001	-1.58	0.001
Pancreatic cancer	6.2. Cancers: Specific types	-1.81	0.000	-1.57	0.001	-1.59	0.001
Breast cancer	6.2. Cancers: Specific types	-1.43	0.019	-1.43	0.001	-1.65	0.000
Prostate cancer	6.2. Cancers: Specific types	-1.4	0.035	-1.67	0.000	-1.65	0.000
Melanoma	6.2. Cancers: Specific types	-1.43	0.033	-1.49	0.008	-1.69	0.000
Renal cell carcinoma	6.2. Cancers: Specific types	-1.5	0.013	-1.69	0.000	-1.72	0.000
Non-small cell lung cancer	6.2. Cancers: Specific types	-1.42	0.028	-1.47	0.007	-1.74	0.000
Endometrial cancer	6.2. Cancers: Specific types	-1.47	0.033	-1.48	0.009	-1.79	0.000
Chronic myeloid leukemia	6.2. Cancers: Specific types	-1.96	0.000	-1.65	0.000	-1.8	0.000
Amphetamine addiction	6.5. Substance dependence	-1.17	0.242	-1.45	0.021	-1.41	0.031
Cocaine addiction	6.5. Substance dependence	-1.31	0.115	-1.5	0.019	-1.6	0.004
Dilated cardiomyopathy	6.6. Cardiovascular diseases	-1.6	0.003	-1.51	0.002	-1.4	0.016
AGE-RAGE signaling pathway in diabetic complications	6.7. Endocrine and metabolic diseases	-1.94	0.000	-1.89	0.000	-1.58	0.000
Cushing syndrome	6.7. Endocrine and metabolic diseases	-1.3	0.072	-1.5	0.000	-1.67	0.000
Insulin resistance	6.7. Endocrine and metabolic diseases	-1.15	0.232	-1.32	0.029	-1.69	0.000
Tuberculosis	6.8. Infectious diseases: Bacterial	-2.07	0.000	-1.72	0.000	-1.35	0.012
Salmonella infection	6.8. Infectious diseases: Bacterial	-1.99	0.000	-1.4	0.000	-1.42	0.000

Bacterial invasion of epithelial cells	6.8. Infectious diseases: Bacterial	-1.94	0.000	-1.46	0.004	-1.46	0.008
Human immunodeficiency virus 1 infection	6.9. Infectious diseases: Viral	-1.75	0.000	-1.38	0.003	-1.23	0.041
Kaposi sarcoma-associated herpesvirus infection	6.9. Infectious diseases: Viral	-1.86	0.000	-1.51	0.000	-1.36	0.003
Human T-cell leukemia virus 1 infection	6.9. Infectious diseases: Viral	-1.99	0.000	-1.58	0.000	-1.38	0.001
Influenza A	6.9. Infectious diseases: Viral	-2	0.000	-1.37	0.005	-1.43	0.003
Human papillomavirus infection	6.9. Infectious diseases: Viral	-1.68	0.000	-1.48	0.000	-1.5	0.000
Human cytomegalovirus infection	6.9. Infectious diseases: Viral	-1.74	0.000	-1.6	0.000	-1.56	0.000
Hepatitis B	6.9. Infectious diseases: Viral	-1.87	0.000	-1.61	0.000	-1.66	0.000
Malaria	6.10. Infectious diseases: Parasitic	-1.92	0.000	-1.76	0.000	-1.39	0.040
Chagas disease	6.10. Infectious diseases: Parasitic	-1.98	0.000	-1.52	0.001	-1.43	0.006
Leishmaniasis	6.10. Infectious diseases: Parasitic	-2.21	0.000	-1.67	0.000	-1.43	0.012
Toxoplasmosis	6.10. Infectious diseases: Parasitic	-2.12	0.000	-1.59	0.000	-1.45	0.004
Amoebiasis	6.10. Infectious diseases: Parasitic	-2.02	0.000	-1.7	0.000	-1.56	0.000
Endocrine resistance	6.12. Drug resistance: Antineoplastic	-1.49	0.018	-1.48	0.002	-1.59	0.000
EGFR tyrosine kinase inhibitor resistance	6.12. Drug resistance: Antineoplastic	-1.67	0.002	-1.61	0.000	-1.7	0.000
Spinocerebellar ataxia		-0.79	0.867	-1.28	0.036	-1.36	0.014
Viral life cycle - HIV-1		-1.61	0.011	-1.44	0.015	-1.46	0.008
Chemical carcinogenesis - receptor activation		-0.87	0.755	-1.39	0.003	-1.51	0.000
Yersinia infection		-2.12	0.000	-1.55	0.000	-1.56	0.000
Lipid and atherosclerosis		-1.73	0.000	-1.44	0.000	-1.58	0.000
PD-L1 expression and PD-1 checkpoint pathway in cancer		-1.96	0.000	-1.69	0.000	-1.76	0.000
Growth hormone synthesis, secretion and action		-1.49	0.018	-1.72	0.000	-1.87	0.000

frontiers

Supplementary Figure 1

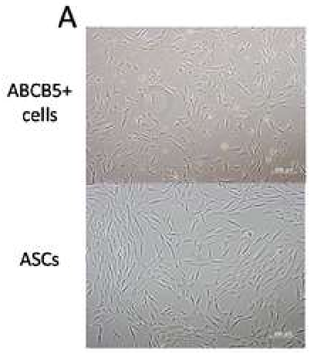

B

Markers	% positivity		MFI values	
	ABCB5+	ASC	ABCB5+	ASC
CD13	100.00 ± 0	100.00 ± 0	24,268 ± 5,558	20,777 ± 4,753
CD29	99.75 ± 0.5	100.00 ± 0	13,542 ± 3,575	18,565 ± 4,841
CD31	1.25 ± 0.55	0.37 ± 0.22	6 ± 22	10 ± 64
CD34	0.40 ± 0.17	0.61 ±0.47	2 ± 5	6 ± 32
CD44	99.93 ± 0.1	100.00 ± 0	10,874 ± 4109	12,144 ± 2,722
CD45	1.92 ± 1.7	0.97 ± 0.84	27 ± 49	36 ± 131
CD49a	73.38 ± 11.91	91.26 ± 8.61	559 ± 113	1,535 ± 418
CD49b	**99.30 ± 0.64**	**77.58 ± 8.53**	**6,662 ± 838**	**1,264 ± 528**
CD49d	85.05 ± 10.62	99.64 ± 0.8	**4,377 ± 578**	**14,540 ± 6175**
CD49e	99.95 ± 0.06	100.00 ± 0.8	**10,384 ± 2,566**	**20,115 ± 5101**
CD49f	50.60 ± 22.13	30.58 ± 18.23	560 ± 441	342 ± 331
CD73	99.93 ± 0.1	100.00 ± 0	53,339 ± 9,900	32,480 ± 15,443
CD90	99.63 ± 0.45	100.00 ± 0	15,280 ± 10,584	37,816 ± 8,790
CD105	91.65 ± 11.73	41.49 ± 53.44	12,124 ± 8,693	13,747 ± 17,521
CD106	1.19 ± 0.6	0.39 ± 0.24	4 ± 23	15 ± 66
CD140a	12.58 ± 8.12	26.45 ± 47.98	84 ± 103	54 ± 74
CD140b	86.08 ± 12.19	79.76 ± 44.09	**1,571 ± 612**	**4,297 ± 1,058**
CD146	7.43 ± 1.52	18.05 ± 13.58	52 ± 36	115 ± 114
CD202b	4.59 ± 3.41	7.56 ± 1.07	122 ± 237	142 ± 141
CD248	**28.60 ± 13.43**	**60.52 ± 21.82**	157 ± 64	477 ± 199
HLA ABC	**88.35 ± 9.03**	**100.00 ± 0**	**4,690 ± 1,240**	**21,923 ± 13,658**
HLA-DR	0.37 ± 0.31	0.77 ± 0.62	2 ± 7	19 ± 6
Integin beta7	0.36 ± 0.33	0.30 ± 0.24	4 ± 4	2 ± 23
NG-2	**57.23 ± 17.57**	**1.63 ± 2.24**	**894 ± 204**	**27 ± 53**

Supplementary Figure 1. Morphology and immunophenotyping of ABCB5+ cells and ASCs

(A): Representative cell culture images (scale bar 200 µm) of ABCB5+ cells (top picture) and ASCs (bottom picture); **(B)**: Surface marker expression of ABCB5+ cells and ASCs measured in flow cytometry, % positivity and MFI = mean fluorescence intensity. Data are shown as mean ± SD N=4),

significant differences indicated in bold: *p<0.5.

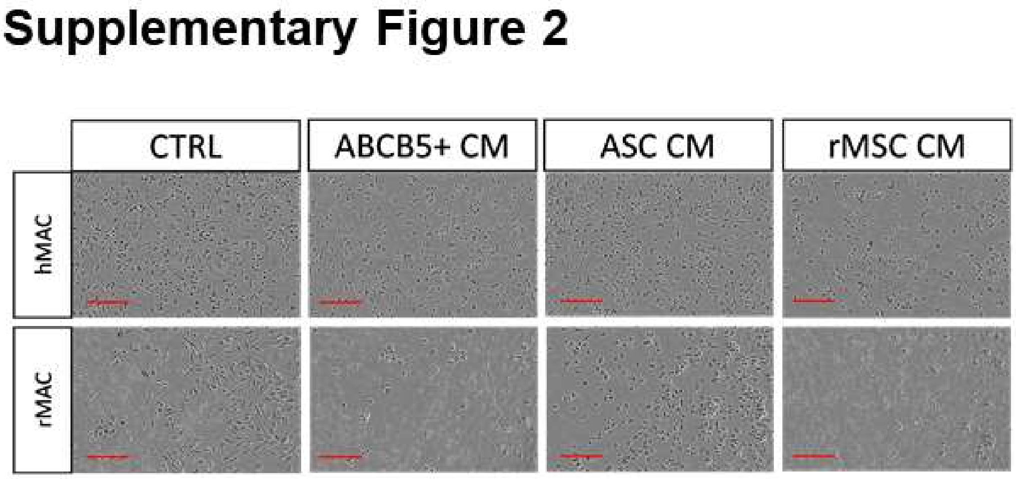

Supplementary Figure 2. Representative images of hMAC and rMAC treated with CM from ABCB5+, ASC and rMSC

Human and rat monocytes were seeded in the presence of CM from ABCB5+, ASC and rMSC supplemented by MCSF 10ng/ml for 6 days until they matured into macrophages. Scale bars 200 µm.

Supplementary Figure 3

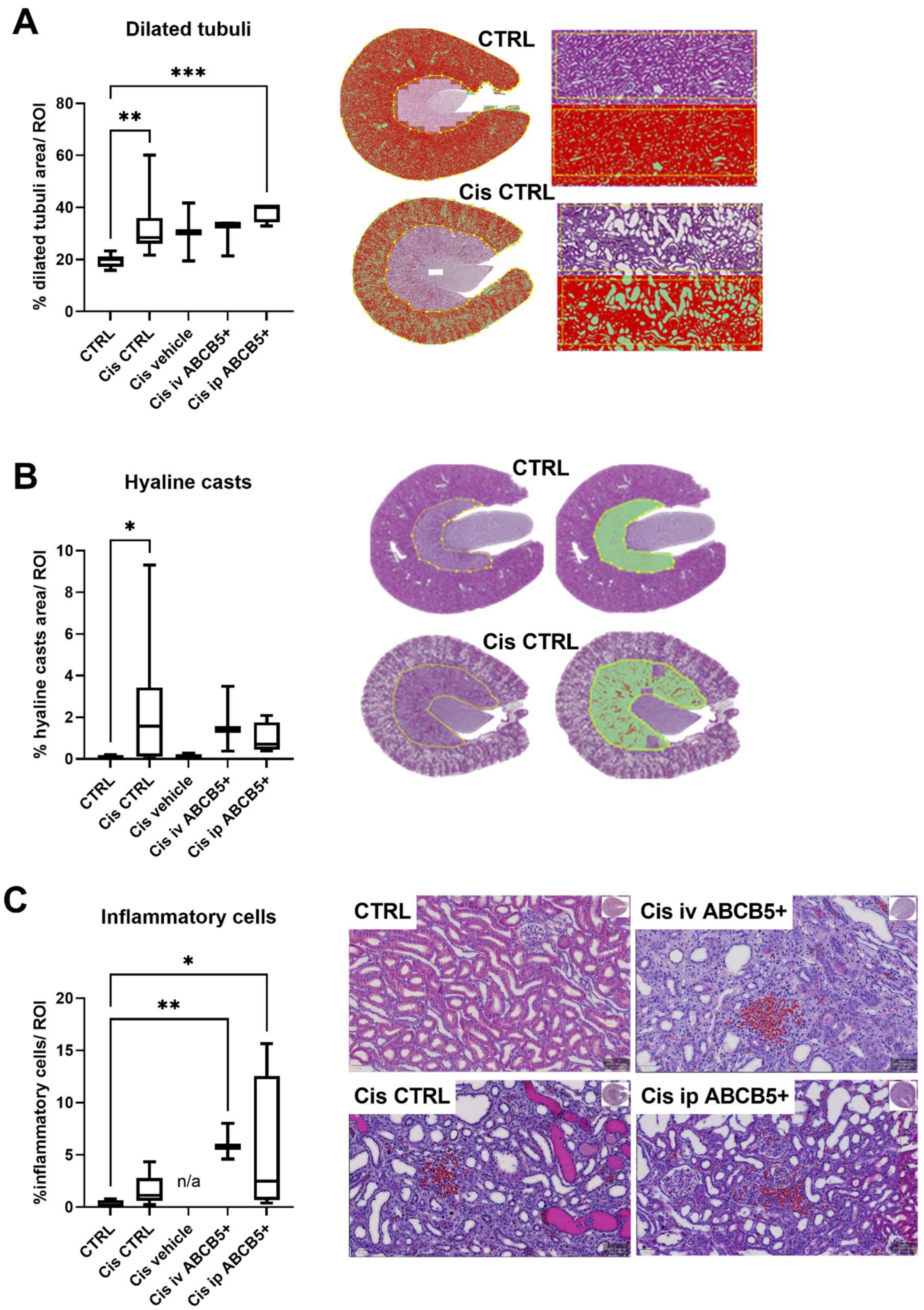

Supplementary Figure 3. Image analysis of histology slides

Image analysis using QuPath. (A) Dilated tubuli and (B) hyaline casts detected by a pixel classifier and (C) inflammatory cells quantified by a trained Random Trees object classifier on regions of interest (ROIs) from adrenal cortex. healthy CTRL N=7, cisplatin CTRL N=16 (N= 14 for inflammatory infiltrates), cisplatin vehicle CTRL N= 3 (infiltrate calculation gave no conclusive results), cisplatin i.v. ABCB5+ N=3, cisplatin i.p. ABCB5+ N=4.
