# Supplementary figures and images for: Clinical-grade human skin-derived ABCB5+ mesenchymal stromal cells exert anti-apoptotic and anti-inflammatory effects in vitro and modulate mRNA expression in a cisplatin-induced kidney injury murine model
Source: Front Immunol. 2024 Jan 11;14:1228928. doi: 10.3389/fimmu.2023.1228928 (PMC10808769; doi:10.3389/fimmu.2023.1228928)

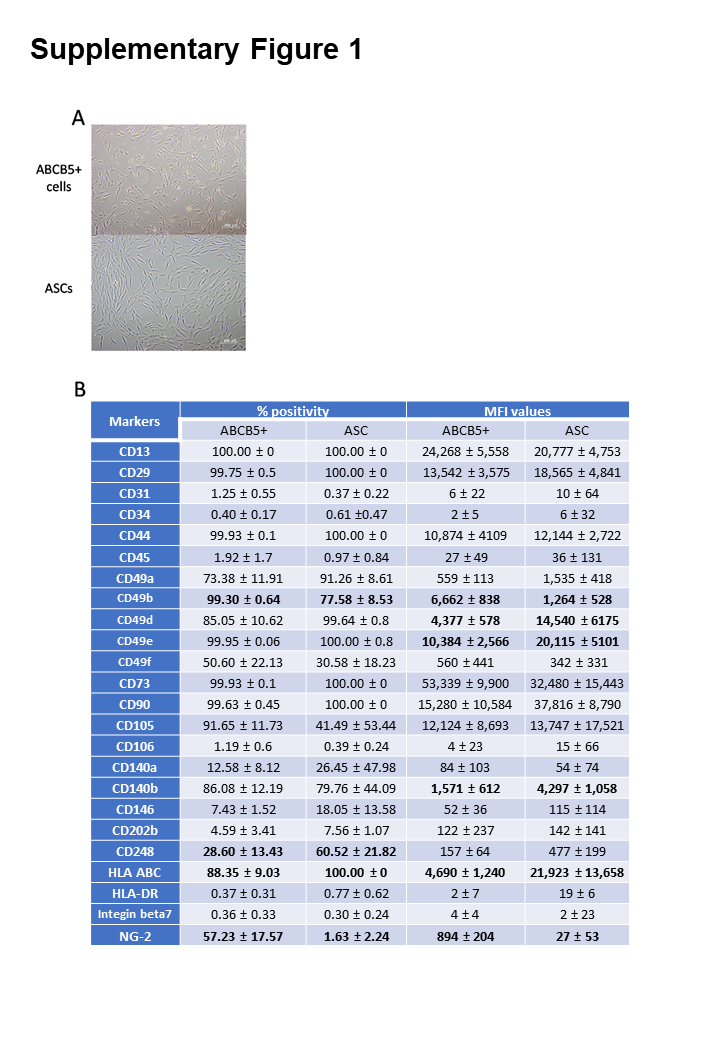

Supplement: Supplementary file 2 [file Image_1.tif]

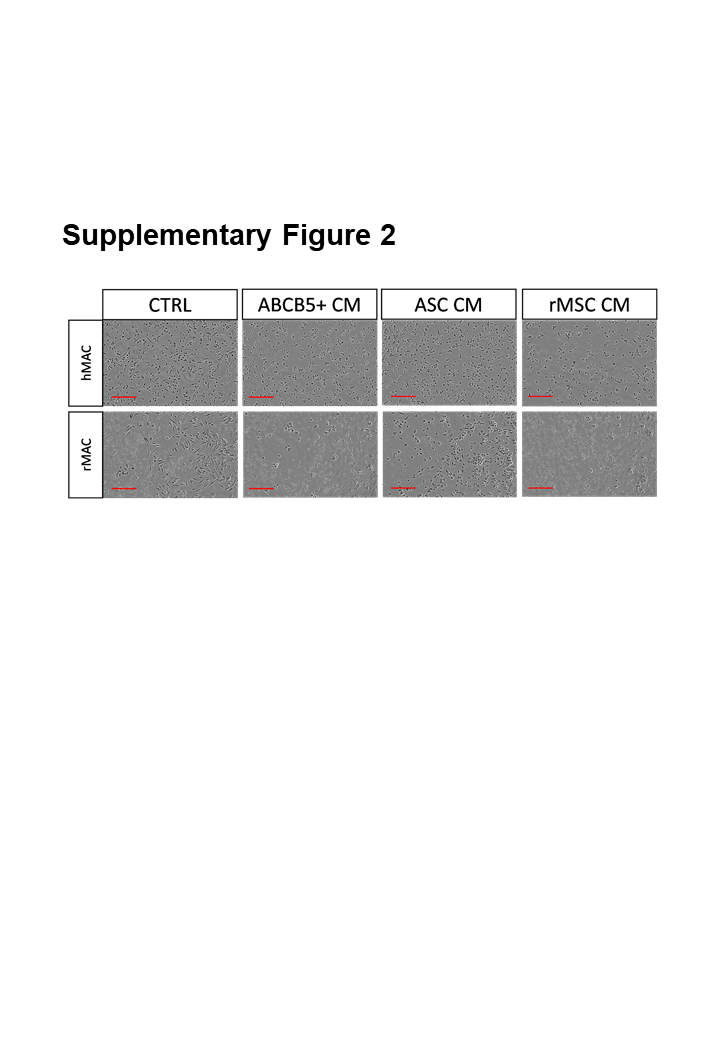

Supplement: Supplementary file 3 [file Image_2.tif]

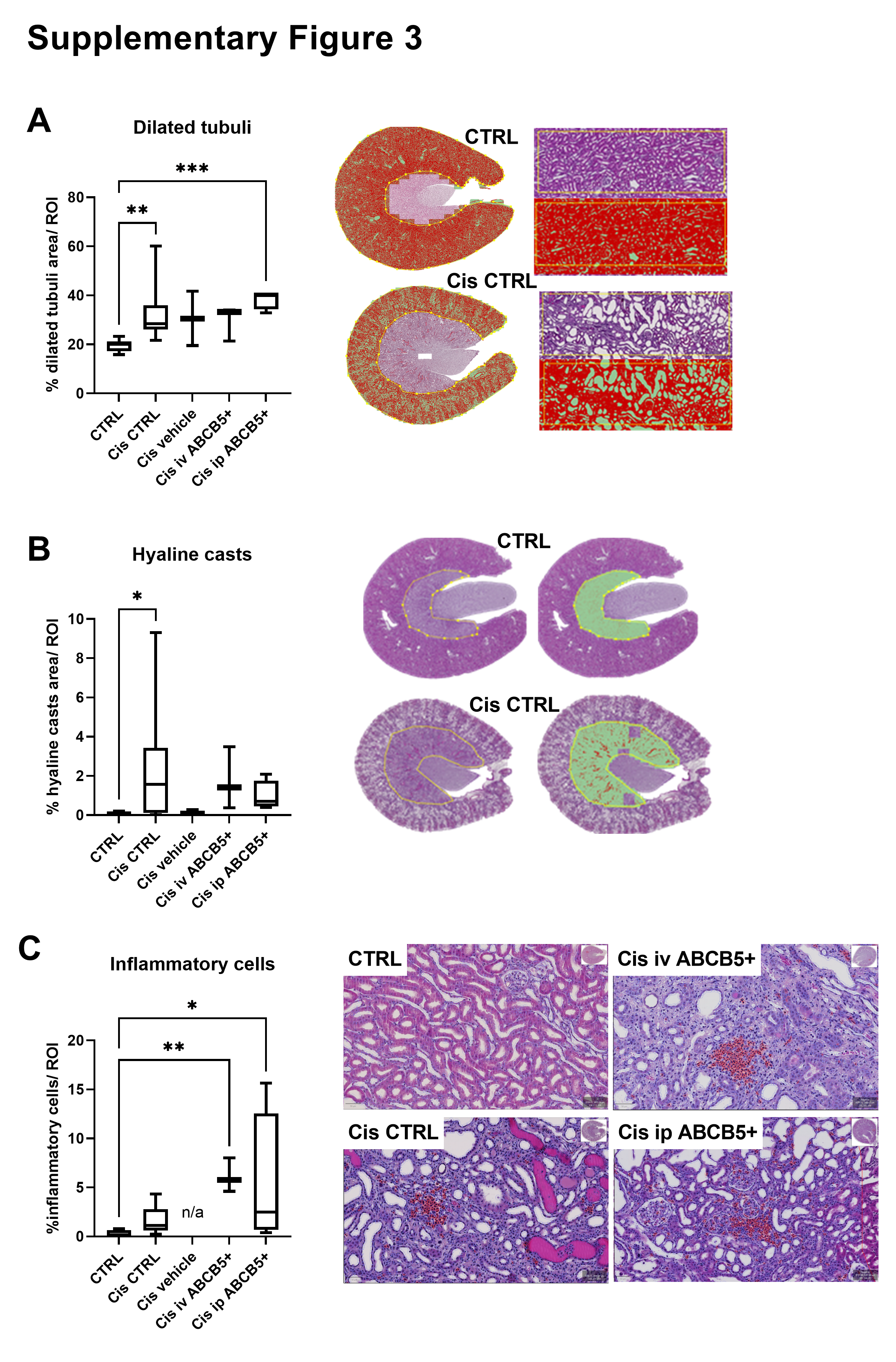

Supplement: Supplementary file 4 [file Image_3.tif]
